# A new order-theoretic characterisation of the polytime computable functions^☆^

**DOI:** 10.1016/j.tcs.2015.03.003

**Published:** 2015-06-20

**Authors:** Martin Avanzini, Naohi Eguchi, Georg Moser

**Affiliations:** Institute of Computer Science, University of Innsbruck, Austria

**Keywords:** Term rewriting, Complexity analysis, Implicit computational complexity, Automation

## Abstract

We propose a new order-theoretic characterisation of the class of polytime computable functions. To this avail we define the *small polynomial path order* (${\mathrm{sPOP}}^{⁎}$ for short). This termination order entails a new syntactic method to analyse the innermost runtime complexity of term rewrite systems fully automatically: for any rewrite system compatible with ${\text{sPOP}}^{⁎}$ that employs recursion up to depth *d*, the (innermost) runtime complexity is polynomially bounded of degree *d*. This bound is tight. Thus we obtain a direct correspondence between a syntactic (and easily verifiable) condition of a program and the asymptotic worst-case complexity of the program.

## 1 Introduction

In this paper we are concerned with the complexity analysis of term rewrite systems (TRSs for short). Based on a careful investigation into the principle of *predicative recursion* as proposed by Bellantoni and Cook [1] we introduce a new termination order, the *small polynomial path order* (${\mathrm{sPOP}}^{⁎}$ for short). The order ${\text{sPOP}}^{⁎}$  provides a new characterisation of the class FP of polytime computable functions. Any function *f* computable by a TRS R compatible with ${\text{sPOP}}^{⁎}$ is polytime computable. On the other hand for any polytime computable function *f*, there exists a TRS $R_{f}$ computing *f* such that R is compatible with ${\text{sPOP}}^{⁎}$. Moreover ${\text{sPOP}}^{⁎}$ directly relates the depth of recursion of a given TRS to the polynomial degree of its runtime complexity. More precisely, we call a rewrite system R *predicative recursive of degree d* if R is compatible with ${\text{sPOP}}^{⁎}$ and the depth of recursion of all function symbols in R is bounded by *d* (see Section 3 for the formal definition). We establish that any predicative recursive rewrite system of degree *d* admits runtime complexity in $O(n^{d})$. Here *n* refers to the sum of the sizes of inputs. Furthermore we obtain a novel, order-theoretic characterisation of $\mathrm{DTIME}(n^{d})$, the class of functions computed on register machines in $O(n^{d})$ steps.

Thus we obtain a direct correspondence between a syntactic (and easily verifiable) condition of a program and the asymptotic worst-case complexity of the program. In this sense our work is closely related to similar studies in the field of *implicit computational complexity* (*ICC* for short). On the other hand the order ${\text{sPOP}}^{⁎}$ entails a new syntactic criteria to automatically establish polynomial runtime complexity of a given TRS. This criteria extends the state of the art in runtime complexity analysis as it is more precise or more efficient than related techniques. Note that the proposed syntactic method to analyse the (innermost) runtime complexity of rewrite systems is fully automatic. For any given TRS, compatibility with ${\text{sPOP}}^{⁎}$ can be efficiently checked by a machine. Should this check succeed, we get an asymptotic bound on the runtime complexity directly from the parameters of the order. It should perhaps be emphasised that compatibility of a TRS with ${\text{sPOP}}^{⁎}$ implies termination and thus our complexity analysis technique does not presuppose termination.

In sum, in this work we make the following contributions:

– We propose a new *recursion-theoretic characterisation* $B_{\mathrm{wsc}}$ over binary words of the class FP. We establish that those $B_{\mathrm{wsc}}$ functions that are definable with *d* nestings of predicative recursion can be computed by predicative recursive TRSs of degree *d* (cf. Theorem 4). Note that these functions belong to $\mathrm{DTIME}(n^{d})$.
– We propose the new termination order ${\text{sPOP}}^{⁎}$; ${\text{sPOP}}^{⁎}$  captures the recursion-theoretic principles of the class $B_{\mathrm{wsc}}$. Thus we obtain a new *order-theoretic characterisation* of the class FP. Moreover, for any predicative recursive TRS of degree *d* its runtime complexity lies in $O(n^{d})$ (cf. Theorem 1). Furthermore this bound is tight, that is, we provide a family of TRSs, delineated by ${\text{sPOP}}^{⁎}$, whose runtime complexity is bounded from below by $\Omega (n^{d})$, cf. Example 5.
– We extend upon ${\text{sPOP}}^{⁎}$ by proposing a generalisation, denoted ${\text{sPOP}}_{\mathrm{PS}}^{⁎}$, admitting the same properties as above. This generalisations incorporates a more general recursion scheme that makes use of *parameter substitution* (cf. Theorem 2).
– We establish a novel, order-theoretic characterisation of $\mathrm{DTIME}(n^{d})$. We show that $\mathrm{DTIME}(n^{d})$ corresponds to the class of functions computable by *tail-recursive* predicative TRSs of degree *d*. This characterisation is based on the generalised small polynomial path order ${\text{sPOP}}_{\mathrm{PS}}^{⁎}$  (cf. Theorem 6).
– ${\text{sPOP}}^{⁎}$ gives rise to a new syntactic method for *polynomial runtime complexity method*. This method is fully automatic. We have implemented the order ${\text{sPOP}}^{⁎}$ in the *Tyrolean Complexity Tool* , version 2.0, an open source complexity analyser [2]. The experimental evidence obtained indicates the efficiency of the method and the obtained increase in precision.

### 1.1 Related work

There are several accounts of predicative analysis of recursion in the (ICC) literature. We mention only those related works which are directly comparable to our work. See [3] for an overview on ICC.

The class $B_{\mathrm{wsc}}$ is a syntactic restriction of the recursion-theoretic characterisation N of the class FEXP of *exponential time computable functions*, given by Arai and the second author in [4]. To account for the fact that FEXP is *not closed* under composition in general, the definition of N relies on a syntactically restricted form of composition. The same composition scheme allows a fine-grained control in our class $B_{\mathrm{wsc}}$ through the degree of recursion. In [5] the authors use the class N as a sufficient basis for an order-theoretic account of FEXP, the *exponential path order* (${\text{EPO}}^{⁎}$ for short). Due to the close relationship of $B_{\mathrm{wsc}}$ and N, our order is both conceptually and technically close to ${\text{EPO}}^{⁎}$.

Notably the clearest connection of our work is to Marion's *light multiset path order* (*LMPO* for short) [6] and the *polynomial path order* (${\text{POP}}^{⁎}$ for short) [7–9]. Both orders form a strict extension of ${\text{sPOP}}^{⁎}$, but lack the precision of the latter. Although LMPO characterises FP, the runtime complexity of compatible TRSs is not polynomially bounded in general. ${\text{POP}}^{⁎}$ induces polynomial runtime complexities, but the obtained complexity certificate is usually very imprecise. In particular, due to the multiset status underlying ${\text{POP}}^{⁎}$, for each d∈N one can form a TRS compatible with ${\text{POP}}^{⁎}$  that defines only a single function, but whose runtime is bounded from below by a polynomial of degree *d*, in the sizes of the inputs.

In Bonfante et al. [10] restricted classes of polynomial interpretations are studied that can be employed to obtain polynomial upper bounds on the runtime complexity of TRSs. Polynomial interpretations are complemented with quasi-interpretations in [11], giving rise to alternative characterisations of complexity classes. None of the above results are applicable to relate the depth of recursion to the runtime complexity, in the sense mentioned above. Furthermore it is unknown how the body of work on quasi-interpretations can be employed in the context of runtime complexity analysis. We have also drawn motivation from Leivant's and Marion's characterisations of $\mathrm{DTIME}(n^{d})$ [12,13], that provide related fine-grained classification of the polytime computable functions. Again, these results lack applicability in the context of runtime complexity analysis.

Polynomial complexity analysis is an active research area in rewriting. Starting from [14] interest in this field greatly increased over the last years, see for example [15–18] and [19] for an overview. This is partly due to the incorporation of a dedicated category for complexity into the annual termination competition (TERMCOMP).^1^ However, it is worth emphasising that the most powerful techniques for runtime complexity analysis currently available, basically employ semantic considerations on the rewrite systems, which are notoriously inefficient.

We also want to mention ongoing approaches for the automated analysis of resource usage in programs. Notably, Hoffmann et al. [20] provide an automatic multivariate amortised cost analysis exploiting typing, which extends earlier results on amortised cost analysis. Finally Albert et al. [21] present an automated complexity tool for Java Bytecode programs, Alias et al. [22] give a complexity and termination analysis for flowchart programs, and Gulwani et al. [23] as well as Zuleger et al. [24] provide an automated complexity tool for C programs.

### 1.2 Outline

We present the main intuition behind ${\text{sPOP}}^{⁎}$ and provide an informal account of the obtained technical results.

The order ${\text{sPOP}}^{⁎}$ essentially embodies the predicative analysis of recursion set forth by Bellantoni and Cook [1]. In [1] a recursion-theoretic characterisation B of the class of polytime computable functions is proposed. This analysis is connected to the important principle of *tiering* introduced by Simmons [25] and Leivant [26,27,12]. The essential idea is that the arguments of a function are separated into *normal* and *safe* arguments (or correspondingly into arguments of different tiers). Building on this work we present a subclass $B_{\mathrm{wsc}}$ of B. Crucially the class $B_{\mathrm{wsc}}$ admits only a weak form of composition. Inspired by a result of Handley and Wainer [28], we show that $B_{\mathrm{wsc}}$ captures the polytime functions. This establishes our first main result.

**Fig. 1:**
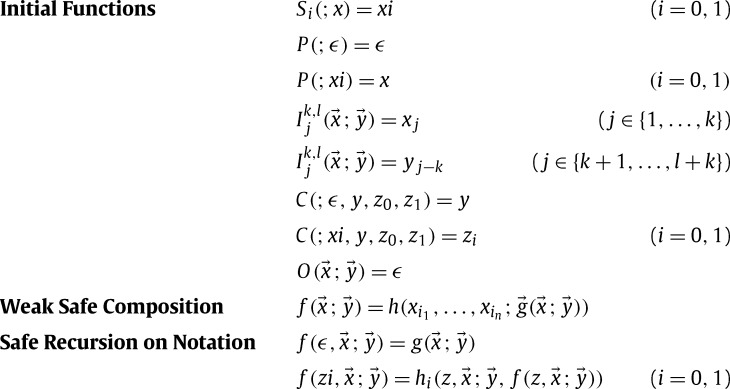
Defining initial functions and operations for $B_{\mathrm{wsc}}$.

We formulate the class $B_{\mathrm{wsc}}$ over the set ${\left\{0,1\right\}}^{⁎}$ of binary words, the empty word is denoted by *ϵ*. Arguments of functions are partitioned into normal and safe ones. In notation, we write $f(t_{1},\ldots ,t_{k};t_{k+1},t_{k+l})$ where *normal* arguments are to the left, and *safe* arguments to the right of the semicolon. Abbreviate $\overset{\to }{x}=x_{1},\ldots ,x_{k}$ and $\overset{\to }{y}=y_{1},\ldots ,y_{l}$. The class $B_{\mathrm{wsc}}$, depicted in Fig. 1, is the smallest class containing certain initial functions and closed under *safe recursion on notation* (**SRN**) and *weak safe composition* (**WSC**). By the weak form of composition only values are ever substituted into normal argument positions.

Suppose the definition of a TRS R is based on the equations in $B_{\mathrm{wsc}}$. It is not difficult to deduce a precise bound on the runtime complexity of R by measuring the number of nested applications of safe recursion, the so called *depth of recursion*. In contrast Bellantoni and Cooks definition [1] of B is obtained from Fig. 1 by replacing weak safe composition with the more liberal scheme of *safe composition* (**SC**): $f(\overset{\to }{x}\;;\;\overset{\to }{y})=h(\overset{\to }{i}(\overset{\to }{x}\;;\;)\;;\;\overset{\to }{j}(\overset{\to }{x}\;;\;\overset{\to }{y}))$. As soon as one of the functions $\overset{\to }{i}$ is size increasing, a tight correspondence between the runtime complexity and the depth of recursion is lost.

Our central observation is that from the function algebra $B_{\mathrm{wsc}}$, one can distill a termination argument for the TRS R. With ${\text{sPOP}}^{⁎}$, this implicit termination argument is formalised as a termination order. In order to employ the separation of normal and safe arguments, we fix for each defined symbol in R a partitioning of argument positions into *normal* and *safe* positions. For constructors we fix (as in $B_{\mathrm{wsc}}$) that all argument positions are safe. Moreover ${\text{sPOP}}^{⁎}$ restricts recursion to normal argument. Dual, only safe argument positions allow the substitution of recursive calls. Via the order constraints we can also guarantee that only normal arguments are substituted at normal argument positions. We emphasise that our notion of predicative recursive TRS is more liberal than the class $B_{\mathrm{wsc}}$. Notably values are not restricted to words, but can be formed from arbitrary constructors. We allow arbitrary deep right-hand sides, and implicit casting from normal to safe arguments. Still the main principle underlying $B_{\mathrm{wsc}}$ remains reflected.

The remainder of the paper is organised as follows. After giving some preliminaries, Section 3 introduces the order ${\text{sPOP}}^{⁎}$. Here we also prove correctness of ${\text{sPOP}}^{⁎}$  with respect to runtime complexity analysis. In Section 4 we incorporate parameter substitution into the order ${\text{sPOP}}^{⁎}$. In Section 5 we then show that these orders are complete for FP, in particular we precisely relate ${\text{sPOP}}^{⁎}$ to the class $B_{\mathrm{wsc}}$. In total we obtain an order-theoretic characterisation of FP. Exploiting the fine-grained control given by the degree of recursion, in Section 6 we provide an order-theoretic characterisation of $\mathrm{DTIME}(n^{d})$. Finally in Sections 7 and 8 we clarify the expressiveness of the established small polynomial path orders and conclude.

## 2 Preliminaries

We denote by N the set of natural numbers {0,1,2,…}. For a finite *alphabet* A of *characters*, we denote by W(A) the set of *words over* A, the empty word is denoted by *ε*. Let *R* be a binary relation. We denote by $R^{+}$ the transitive, by $R^{⁎}$ the transitive and reflexive closure, and $R^{n}$ denotes for n∈N the *n*-fold composition of *R*. We write *aRb* for (a,b)∈R, the relation *R* is *well-founded* if there exists no infinite sequence $a_{1}Ra_{2}Ra_{3}R\ldots$. The relation *R* is a *preorder* if it is transitive and reflexive, it is a *strict partial order* if it is irreflexive, antisymmetric and transitive, and *R* is an equivalence relation if it is reflexive, symmetric and transitive. Note that the transitive and reflexive closure of an order *R* (on a set *S*) gives always a preorder. Consider a preorder ⩾. Define a∼b if a⩾b and b⩾a. Then this equivalence defines a partitioning of ⩾ into the equivalence ∼ and a strict partial order >.

### 2.1 Term rewriting

We assume at least nodding acquaintance with the basics of term rewriting [29]. We fix a countably infinite set of *variables* V and a finite set of *function symbols* F, the *signature*. For each f∈F, the *arity* of *f* is fixed. The set of terms formed from F and V is denoted by T(F,V). A term t∈T(F,V) is called *ground* if it contains no variables. The set of ground terms is indicated by T(F). The signature F contains a distinguished set of *constructors* C⊆F, elements of T(C)⊆T(F) are called *values*. Elements of F that are not constructors are called *defined symbols* and collected in D. If not mentioned otherwise we denote by x,y,z variables, f,g,h,… denote defined symbols. Terms are denoted by l,r or s,t, and values by u,v,w. All denotations are possibly followed by subscripts. We use the notation $\overset{\to }{s}$ to abbreviate a finite sequence of terms $s_{1},\ldots ,s_{n}$.

The root symbol of term *t* is denoted as rt(t). The *size* of *t* is denoted by |t| and refers to the number of occurrences of symbols *t*, the depth dp(t) is given recursively by dp(t)=1 if t∈V, and $\mathrm{dp}(f(t_{1},\ldots ,t_{n}))=1+\max\{\mathrm{dp}(t_{i})|i=1,\ldots ,n\}$. Here we employ the convention that the maximum of an empty set is equal to 0. A *rewrite rule* is a pair (l,r) of terms, in notation l→r, such that the *left-hand* side $l=f(l_{1},\ldots ,l_{n})$ is not a variable, the *root f* is defined, and all variables appearing in the *right-hand r* occur also in *l*. A *term rewrite system* (*TRS* for short) R is a set of rewrite rules.

We adopt *call-by-value* semantics and define the *rewrite relation* $\to_{R}$ as follows.

$$\text{(i)}\;\frac{f(l_{1},\ldots ,l_{n})\to r\in R,\;\sigma \;:\;V\to T(C)}{f(l_{1}\sigma ,\ldots ,l_{n}\sigma )\to_{R}r\sigma }\;\text{(ii)}\;\frac{s\to_{R}t}{f(\ldots ,s,\ldots )\to_{R}f(\ldots ,t,\ldots )}.$$

If $s\to_{R}t$ we say that *s reduces to t* in one step. For (i) we make various assumptions on R: we suppose that there is exactly one *matching* rule $f(l_{1},\ldots ,l_{n})\to r\in R$; the arguments $l_{i}$ (i=1,…,n) contains no defined symbols; and variables occur only once in $f(l_{1},\ldots ,l_{n})$. That is, throughout this paper we fix R to denote a *completely defined*,^2^ *orthogonal constructor* TRS [29]. Furthermore we are only concerned with *innermost* rewriting. Note that orthogonality enforces that our model of computation is deterministic.^3^ If a term *t* has a normal form, then this term is unique and denoted by *t*↓. For every *n*-ary defined symbol f∈D, R defines a partial function $〚f〛\;:\;T{\left(C\right)}^{n}\to T(C)$ where

$$〚f〛(u_{1},\ldots ,u_{n}):=f(u_{1},\ldots ,u_{n})↓\;\text{if}\;f(u_{1},\ldots ,u_{n})↓\in T(C),$$

and $〚f〛(u_{1},\ldots ,u_{n})$ is undefined otherwise. Note that when R is *terminating*, i.e. when $\to_{R}$ is well-founded, the function 〚f〛 is total.

Following [30] we adopt a unitary cost model. Bounds are of course expressed with respect to the size of terms. Let $T_{b}(F)$ denote the set of *basic* (also called *constructor based*) terms $f(u_{1},\ldots ,u_{n})$ where f∈D and $u_{1},\ldots ,u_{n}\in T(C)$. We define the *(innermost) runtime complexity function* ${\mathrm{rc}}_{R}\;:\;N\to N$ as

$${\mathrm{rc}}_{R}(n):=\max\{\ell {|\exists s\in T_{b}(F),{|s{|⩽n\;\text{and}\;s=t_{0}\to }_{R}t_{1}\to }_{R}\cdots \to }_{R}t_{\ell }\}.$$

Hence ${\mathrm{rc}}_{R}(n)$ maximises over the *derivation height* of terms *s* of size up to *n*, regarding only basic terms. The latter restriction accounts for the fact that computations start only from basic terms. The runtime complexity function is well-defined if R is terminating. If ${\mathrm{rc}}_{R}$ is asymptotically bounded from above by a polynomial, we simply say that the runtime of R is polynomially bounded. This unitary cost model is reasonable:

Proposition 1 **Adequacy theorem**

*(See* *[31–33]**.) All functions* 〚f〛 *computed by* R *are computable on a conventional models of computation, viz Turing machines, such that the time complexity on the latter is polynomially related to* ${\mathrm{rc}}_{R}$*.*

In particular, if the runtime of R is polynomially bounded then 〚f〛 is polytime computable on a Turing machine for all f∈D.

We say that a function symbol *f* is *defined based on g*, in notation $f{►}_{R}g$, if there exists a rewrite rule $f(l_{1},\ldots ,l_{n})\to r\in R$ where *g* occurs in *r*. We call *f recursive* if $f{►}_{R}^{+}f$ holds, i.e. if *f* is defined based on itself. Recursive functions symbols are collected in $D_{\mathrm{rec}}\subseteq D$. Noteworthy our notion also captures mutual recursion. We denote by ⩾ the least preorder on F containing ${►}_{R}$ and where constructors are equivalent, i.e. c⩾d and d⩾c for all constructors c,d∈C. The preorder ⩾ is called the *precedence* of R. We denote by > and ∼ the separation of ⩾ into the strict partial order > and the equivalence ∼. Note that for f∼g, if f∈C then also g∈C; similar $f\in D_{\mathrm{rec}}$ implies $g\in D_{\mathrm{rec}}$. The *rank* of f∈F with respect to ⩾ is inductively defined by rk(f)=1+max⁡{rk(g)|f>g}. The *depth of recursion* rd(f) of f∈F is defined in correspondence to the rank, but only takes recursive symbols into account: let d=max⁡{rd(g)|f>g} be the maximal recursion depth of a function symbol *g* underlying the definition of *f*; then rd(f):=1+d if *f* is recursive, otherwise rd(f):=d.

Example 1 Consider following TRS $R_{\mathrm{arith}}$, written in predicative notation.

$$\begin{array}{ccc} 1:\;+(0;y)\to y & 3:\;+(s(;x);y)\to s(+(x;y)) & 5:\;f(x,y;)\to +(x;\times (y,y;))\\ 2:\;\times (0,y;)\to 0 & 4:\;\times (s(;x),y;)\to +(y;\times (x,y;)). & \end{array}$$

The TRS $R_{\mathrm{arith}}$ follows along the line of $B_{\mathrm{wsc}}$ from Fig. 1. The functions 〚+〛 and 〚×〛 denote addition and multiplication on natural numbers, in particular $〚f〛(s^{m}(0),s^{n}(0))=s^{r}(0)$ where $r=m+n^{2}$. The precedence is given by f>×>+>s∼0 where addition (+) and multiplication (×) are recursive, but f is not recursive. We have rd(+)=1 since addition is recursive, as f is not recursive but multiplication is recursive we have rd(f)=rd(×)=2.

### 2.2 Register machines

In this paper, we are considering *register machine* (*RM* for short) over words W(A) as initially proposed by Shepherdson and Sturgis in [34]. We employ following notations and conventions. An RM *M* consists of a *finite* set of *registers* that store words over W(A). Like values, i.e. constructor terms, words are denoted by u,v,w, and $\overset{\to }{u},\overset{\to }{v},\overset{\to }{w}$ denote sequences of words. No confusion can arise from this. For *r* a register, we use 〈r〉 to refer to the content of register *r*. The *control* of *M* consists of a finite sequences of (labeled) *instructions* $I_{1};I_{2};\ldots ;I_{l}$ which are executed sequentially by *M*. Here an instruction can be one of the following:

(i) *Append instruction* $A^{\left(a\right)}(r)$: place a∈A on the left-hand end of 〈r〉;
(ii) *Delete instruction* D(r): remove the left-most character from 〈r〉, if 〈r〉≠ε;
(iii) *Conditional jump instruction* $J^{\left(a\right)}(r)[j]$: jump to instruction $I_{j}$, if the left-most character of 〈r〉 is a∈A, otherwise proceed with the next instruction;
(iv) *Copy instruction* $C(r,r^{\prime })$: overwrite $\langle r^{\prime }\rangle$ by 〈r〉.

Our definition departs from [34] in following minor respects. Unlike in [34], we suppose that the set of registers is finite. This simplification does not impose any restrictions. Due to the absence of memory indirection instructions, only a fixed number of registers can be accessed by a machine *M* anyway. The instructions (i)–(iii) correspond to the *minimal* instruction set given in [34, Section 6], with the difference that in [34] the instruction (i) appends to the right. The additional copy instruction (iv) added from the extended instruction set of [34, Section 2] ensures that copying words has unitary cost. A configuration of the RM *M* is a tuple $\langle j,w_{1},\ldots ,w_{m}\rangle$ where $w_{1},\ldots ,w_{m}\in W(A)$ are the content of the *m* registers and *j* ranges over the *labels* 1,…,l of instructions $I_{1},\ldots ,I_{l}$ of *M*, and the dedicated *halting label* l+1. We denote by $\to_{M}$ the one-step transition relation obtained in the obvious way from our informal account of the instruction set (i)–(iv). For the halting label l+1 we set $\langle l+1,u_{1},\ldots ,u_{m}\rangle \to_{M}\langle l+1,u_{1},\ldots ,u_{m}\rangle$ for all words $u_{i}$ (i=1,…,m). We say that the RM *M computes* the (partial) function $f_{M}\;:\;W{\left(A\right)}^{k}\to W(A)$ with k⩽m defined as follows:

$$f_{M}(u_{1},\ldots ,u_{k}):=v_{m}\;:\Leftrightarrow \;\exists \ell .\langle 1,u_{1},\ldots ,u_{k},\overset{\to }{\varepsilon }\rangle \to_{M}^{\ell }\langle l+1,v_{1},\ldots ,v_{k}\rangle .$$

We also say that on inputs $u_{1},\ldots ,u_{k}$ the computation halts in *ℓ* steps. Denote by |u| the length, or *size*, of the word *u*. Extend this to $\overset{\to }{u}=u_{1},\ldots ,u_{k}$ so that $\left|\overset{\to }{u}|=\sum_{i=1}^{k}|u_{i}\right|$ denotes the sum of the sizes of $\overset{\to }{u}$. Let d∈N. We denote by $\mathrm{DTIME}(n^{d})$ the class of functions $f\;:\;W{\left(A\right)}^{k}\to W(A)$ computed by some RM *M* in the above way, where *M* halts on all inputs $\overset{\to }{u}$ in no more than $O({\left|\overset{\to }{u}\right|}^{d})$ steps.

## 3 The small polynomial path order

We arrive at the formal definition of the *small polynomial path order* (${\text{sPOP}}^{⁎}$ for short). Conceptually this order is a tamed recursive path order with product status, embodying *predicative analysis* of recursion set forth by Bellantoni and Cook [1].

Throughout this section, fix a TRS R. For each function symbol *f*, we assume an a priori separation of argument positions into *normal* and *safe* ones. Arguments under normal positions play the role of recursion parameters, whereas safe argument positions allow the substitution of recursive results, compare the definition of $B_{\mathrm{wsc}}$ drawn in Fig. 1 on page 5. For constructors *c* we fix that all argument positions are safe. As in Example 1, we indicate this separation directly in terms and write $f(\overset{\to }{s}\;;\;\overset{\to }{t})$ where the arguments $\overset{\to }{s}$ to the left of the semicolon are normal, the remaining arguments $\overset{\to }{t}$ are safe. This separation and the precedence ⩾ underlying the analysed TRS R *induces* an *instance* of ${\text{sPOP}}^{⁎}$, which is denoted by ${>}_{{\mathrm{spop}}^{⁎}}$ below.

In order to define ${>}_{{\mathrm{spop}}^{⁎}}$, we introduce some auxiliary relations. First of all, we lift equivalence ∼ underlying the precedence ⩾ of R to terms, disregarding the order on arguments: *s* and *t* are *equivalent*, in notation s∼t, if s=t, or $s=f(s_{1},\ldots ,s_{n})$ and $t=g(t_{1},\ldots ,t_{n})$ where f∼g and there exists a permutation *π* on argument positions {1,…,n} such that $s_{i}∼t_{\pi (i)}$ for all i=1,…,n. *Safe equivalence* $\overset{s}{∼}\subseteq ∼$ takes also the separation of argument positions into account. In the definition of $s\overset{s}{∼}t$, we additionally require that *i* is a normal argument position of *f* if and only if π(i) is normal argument position of *g*. We emphasise that ∼ (and consequently $\overset{s}{∼}$) preserves values: if s∼t and s∈T(C) then t∈T(C). We extend the subterm relation to term equivalence. Consider $s=f(s_{1},\ldots ,s_{k};s_{k+1},\ldots ,s_{k+l})$. Define $s⊵{/}_{∼}t$ if either s∼t or $s▷{/}_{∼}t$, where $s▷{/}_{∼}t$ holds if $s_{i}⊵{/}_{∼}t$ for some argument $s_{i}$ of *s* (i=1,…k+l). We denote by ${▷}^{\text{n}}{/}_{∼}$ the restriction of $▷{/}_{∼}$ where only normal arguments are considered: $s{▷}^{\text{n}}{/}_{∼}t$ if $s_{i}⊵{/}_{∼}t$ for some *normal* argument position i∈{1,…,k}.

Definition 1 Let *s* and *t* be terms such that $s=f(s_{1},\ldots ,s_{k};s_{k+1},\ldots ,s_{k+l})$. Then $s{>}_{{\mathrm{spop}}^{⁎}}t$ if one of the following alternatives holds.

1. $s_{i}{⩾}_{{\mathrm{spop}}^{⁎}}t$ for some argument $s_{i}$ of *s* (i∈{1,…,k+l}).
2. f∈D, $t=g(t_{1},\ldots ,t_{m};t_{m+1},\ldots ,t_{m+n})$ where f>g and the following holds:
  – $s{▷}^{\text{n}}{/}_{∼}t_{j}$ for all normal arguments $t_{1},\ldots ,t_{m}$;
  – $s{>}_{{\mathrm{spop}}^{⁎}}t_{j}$ for all safe arguments $t_{m+1},\ldots ,t_{m+n}$;
  – *t* contains at most one (recursive) function symbol *h* with f∼h.
3. $f\in D_{\mathrm{rec}}$, $t=g(t_{1},\ldots ,t_{k};t_{k+1},\ldots ,t_{k+l})$ where f∼g and the following holds:
  – $\langle s_{1},\ldots ,s_{k}\rangle {>}_{{\mathrm{spop}}^{⁎}}\langle t_{\pi (1)},\ldots ,t_{\pi (k)}\rangle$ for some permutation *π* on {1,…,k};
  – $\langle s_{k+1},\ldots ,s_{k+l}\rangle {⩾}_{{\mathrm{spop}}^{⁎}}\langle t_{\tau (k+1)},\ldots ,t_{\tau (k+l)}\rangle$ for some permutation *τ* on {k+1,…,k+l}.

Here $s{⩾}_{{\mathrm{spop}}^{⁎}}t$ denotes that either $s\overset{s}{∼}t$ or $s{>}_{{\mathrm{spop}}^{⁎}}t$ holds. In the last clause we use ${>}_{{\mathrm{spop}}^{⁎}}$ also for the extension of ${>}_{{\mathrm{spop}}^{⁎}}$ to products: $\langle s_{1},\ldots ,s_{n}\rangle {⩾}_{{\mathrm{spop}}^{⁎}}\langle t_{1},\ldots ,t_{n}\rangle$ means $s_{i}{⩾}_{{\mathrm{spop}}^{⁎}}t_{i}$ for all i=1,…,n, and $\langle s_{1},\ldots ,s_{n}\rangle {>}_{{\mathrm{spop}}^{⁎}}\langle t_{1},\ldots ,t_{n}\rangle$ indicates that additionally $s_{i_{0}}{>}_{{\mathrm{spop}}^{⁎}}t_{i_{0}}$ holds for at least one $i_{0}\in \{1,\ldots ,n\}$.

Throughout the following, we write $s{>}_{{\mathrm{spop}}^{⁎}}^{\langle i\rangle }t$ if $s{>}_{{\mathrm{spop}}^{⁎}}t$ follows from the *i*th clause in Definition 1. A similar notation is employed for the consecutive introduced orders.

We say that the TRS R is *compatible* with ${>}_{{\mathrm{spop}}^{⁎}}$ if all rules are *oriented* from left to right: $l{>}_{{\mathrm{spop}}^{⁎}}r$ for all rules l→r∈R. As ${\text{sPOP}}^{⁎}$ forms a restriction of the recursive path order, compatibility with ${\text{sPOP}}^{⁎}$ implies termination [29].

Definition 2 We call the TRS R *predicative recursive (of degree d)* if R is compatible with an instance of ${\text{sPOP}}^{⁎}$ and the maximal recursion depth rd(f) of f∈F is *d*.

Consider the orientation of a rule $f(l_{1},\ldots ,l_{n})\to r\in R$. The case ${>}_{{\mathrm{spop}}^{⁎}}^{\langle 2\rangle }$ is intended to capture functions *f* defined by weak safe composition (**WSC**), compare Fig. 1. In particular the use of ${▷}^{\text{n}}{/}_{∼}$ allows only the substitution of normal arguments of *f* in normal argument positions of *g*. The last restriction put onto ${>}_{{\mathrm{spop}}^{⁎}}^{\langle 2\rangle }$ is used to prohibit multiple recursive calls. If one drops this restriction, the TRS consisting of f(0;)→0 and f(s(;x);)→c(;f(x;),f(x;)) is compatible with ${\text{sPOP}}^{⁎}$ but its runtime complexity can be only exponentially bounded. Finally, ${>}_{{\mathrm{spop}}^{⁎}}^{\langle 3\rangle }$ accounts for recursive calls, in combination with ${>}_{{\mathrm{spop}}^{⁎}}^{\langle 2\rangle }$ we capture safe recursion (**SRN**). The next theorem provides our second main result.

Theorem 1 *Suppose* R *is a predicative recursive TRS of degree d. Then the derivation height of any basic term* $f(\overset{\to }{u}\;;\;\overset{\to }{v})$ *is bounded by a polynomial of degree* rd(f) *in the sum of the depths of normal arguments* $\overset{\to }{u}$*. In particular, the runtime complexity function* ${\mathrm{rc}}_{R}$ *is bounded by a polynomial of degree d.*

As a consequence of Theorem 1 and the adequacy theorem (c.f. Proposition 1), any predicative recursive (and orthogonal) TRS R computes a function from FP. We remark that Theorem 1 remains valid for the standard notion of innermost rewriting [29] on constructor TRSs. Neither orthogonality nor our fixed call-by-value reduction strategy is essential, compare [9].

We continue with an informal account of Definition 1 in our running example, the admittedly technical proof is shortly postponed.

Example 2 **Example 1 continued**

We show that the TRS $R_{\mathrm{arith}}$ depicted in Example 1 is predicative recursive. Recall that the precedence underlying $R_{\mathrm{arith}}$ is given by f>×>+>s∼0, and that $D_{\mathrm{rec}}=\{\times ,+\}$. The degree of recursion of $R_{\mathrm{arith}}$ is thus two.

The case ${>}_{{\mathrm{spop}}^{⁎}}^{\langle 1\rangle }$ is standard in recursive path orders and allows the treatment of projections as in rules 1 and 2. We have $+(0\;;\;y){>}_{{\mathrm{spop}}^{⁎}}^{\langle 1\rangle }y$ using $y\overset{s}{∼}y$ and likewise $\times (0,y\;;\;){>}_{{\mathrm{spop}}^{⁎}}^{\langle 1\rangle }0$ using $0\overset{s}{∼}0$. Observe that the rule

5: f(x,y;)→+(x;×(y,y;)),

is oriented by ${>}_{{\mathrm{spop}}^{⁎}}^{\langle 2\rangle }$ only: using f>× and twice $f(x,y\;;\;){▷}^{\text{n}}{/}_{∼}y$, i.e., *y* is a normal argument of f(x,y;), we have $f(x,y\;;\;){>}_{{\mathrm{spop}}^{⁎}}^{\langle 2\rangle }\times (y,y\;;\;)$. Using that also f>+ and $f(x,y\;;\;){▷}^{\text{n}}{/}_{∼}x$ holds, another application of ${>}_{{\mathrm{spop}}^{⁎}}^{\langle 2\rangle }$ orients rule 5.

Finally, consider the recursive cases of addition (rule 3) and multiplication (rule 4). These can be oriented by a combination of ${>}_{{\mathrm{spop}}^{⁎}}^{\langle 2\rangle }$ and ${>}_{{\mathrm{spop}}^{⁎}}^{\langle 3\rangle }$. We exemplify this on the rule

4: ×(s(;x),y;)→+(y;×(x,y;)).

Employing ×>+, case ${>}_{{\mathrm{spop}}^{⁎}}^{\langle 2\rangle }$ is applicable. Thus orientation of this rule simplifies to $\times (s(\;;\;x),y\;;\;){▷}^{\text{n}}{/}_{∼}y$ and $\times (s(\;;\;x),y\;;\;){>}_{{\mathrm{spop}}^{⁎}}\times (x,y\;;\;)$. The former constraint is satisfied by definition. Since × is recursive, using ${>}_{{\mathrm{spop}}^{⁎}}^{\langle 3\rangle }$ the latter constraint reduces to $\langle s(\;;\;x),y\rangle {>}_{{\mathrm{spop}}^{⁎}}\langle x,y\rangle$ and the trivial constraint $\langle \rangle {⩾}_{{\mathrm{spop}}^{⁎}}\langle \rangle$. Clearly $\langle s(\;;\;x),y\rangle {>}_{{\mathrm{spop}}^{⁎}}\langle x,y\rangle$ holds as $s(\;;\;x){>}_{{\mathrm{spop}}^{⁎}}^{\langle 1\rangle }x$ and $y\overset{s}{∼}y$. Hence we are done.

Note that any other partitioning of argument positions of multiplication invalidates the orientation of rule 4. The sub-constraint $\times (s(\;;\;x),y\;;\;){>}_{{\mathrm{spop}}^{⁎}}\times (x,y\;;\;)$ requires that at least the first argument position of times is normal, the sub-constraint $\times (s(\;;\;x),y\;;\;){▷}^{\text{n}}{/}_{∼}y$ enforces that also the second parameter is normal. The order thus determines that multiplication performs recursion on its first arguments, and that the second parameter should be considered normal since it is used as recursion parameter in addition. Reconsidering the orientation of rule 5, the use of ${▷}^{\text{n}}{/}_{∼}$ propagates that f takes only normal arguments.

By Theorem 1 we obtain that addition admits linear, and multiplication as well as f admits quadratic runtime complexity. Overall the runtime complexity of $R_{\mathrm{arith}}$ is quadratic.

The following examples clarifies the need for data tiering.

Example 3 **Example 2 continued**

Consider the extension of $R_{\mathrm{arith}}$ by the two rules

6:exp(0,y)→s(;0)7:exp(s(;x),y)→×(y,exp(x,y);),

that express exponentiation $y^{x}$ in an exponential number of steps. The definition of exp is not predicative recursive, since the recursive result exp(x,y) is substituted as recursion parameter to multiplication. For this reason the orientation with ${>}_{{\mathrm{spop}}^{⁎}}$ fails.

The next example is negative, in the sense that the considered TRSs admits polynomial runtime complexity, but fails to be compatible with ${\text{sPOP}}^{⁎}$.

Example 4 **Example 3 continued**

Consider now the TRS $R_{\mathrm{arith}}$ where the rule 4 is replaced by the rule

4a: ×(s(;x),y;)→+(×(x,y;);y).

The resulting system admits polynomial runtime complexity. On the other hand, Theorem 1 is inapplicable since the system is not predicative recursive.

We emphasise that the bound provided in Theorem 1 is tight, in the sense that for any *d* we can define a predicative TRS $R_{d}$ of degree *d* admitting runtime complexity $\Omega (n^{d})$.

Example 5 We define a family of TRSs $R_{d}$ (d∈N) inductively as follows: $R_{0}:=\{f_{0}(x\;;\;)\to a\}$ and $R_{d+1}$ extends $R_{d}$ by the rules

$$f_{d+1}(x\;;\;)\to g_{d+1}(x,x\;;\;)\;g_{d+1}(s(\;;\;x),y\;;\;)\to b(\;;\;f_{d}(y\;;\;),g_{d+1}(x,y\;;\;)).$$

Let d∈N. It is easy to see that $R_{d}$ is predicative recursive (with underlying precedence $f_{d}>g_{d}>f_{d-1}>g_{d-1}>\cdots >f_{0}>a∼b$). As only $g_{i}$ (i=1,…,d) are recursive, the recursion depth of $R_{d}$ is *d*.

But also the runtime complexity of $R_{d}$ is in $\Omega (n^{d})$: For d=0 this is immediate. Otherwise, consider the term $f_{d+1}(s^{n}(\;;\;a)\;;\;)$ (n∈N) which reduces to $g_{d+1}(s^{n}(\;;\;a),s^{n}(\;;\;a)\;;\;)$ in one step. As the latter iterates $f_{d}(s^{n}(a))$ for *n* times, the lower bound is established by inductive reasoning.

**Fig. 2:**
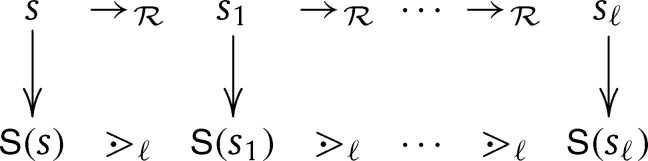
Predicative embedding of $\to_{R}$ into ${⋗}_{\ell }$.

We now show that ${\text{sPOP}}^{⁎}$ is correct, i.e. we prove Theorem 1. Suppose R is a predicative recursive TRS of degree *d*. Our proof makes use of a variety of ingredients. In Definition 3 we define a *predicative interpretation* S that flatten terms to *sequences of terms*, separating safe from normal arguments. In Definition 4 we introduce a family of orders ${\left({⋗}_{\ell }\right)}_{\ell \in N}$ on sequences of terms. The definition of ${⋗}_{\ell }$ (for fixed *ℓ*) does not explicitly mention predicative notions and is conceptually simpler than ${>}_{{\mathrm{spop}}^{⁎}}$. In Lemma 4 we show that predicative interpretations S embeds rewrite steps into ${⋗}_{\ell }$, as pictured in Fig. 2. Consequently the derivation height of *s* is bounded by the length of ${⋗}_{\ell }$ descending sequences, which in turn can be bounded sufficiently whenever *s* is basic (cf. Lemma 7).

Consider a step $C[f(\overset{\to }{u}\sigma \;;\;\overset{\to }{v}\sigma )]\to_{R}C[r\sigma ]=t$. Due to the limitations imposed by ${>}_{{\mathrm{spop}}^{⁎}}$, it is not difficult to see that if *rσ* is not a value itself, then at least all normal arguments are values. We capture this observation in the set $T_{b}^{\to }$, defined as the least set such that (i) $T(C)\subseteq T_{b}^{\to }$, and (ii) if f∈F, $\overset{\to }{v}\subseteq T(C)$ and $\overset{\to }{t}\subseteq T_{b}^{\to }$ then $f(\overset{\to }{v}\;;\;\overset{\to }{t})\in T_{b}^{\to }$. This set is closed under rewriting.

Lemma 1 *Suppose* R *is compatible with* ${>}_{{\mathrm{spop}}^{⁎}}$*. If* $s\in T_{b}^{\to }$ *and* $s\to_{R}t$ *then* $t\in T_{b}^{\to }$*.*

Proof The lemma follows by a straightforward inductive argument on Definition 1.  □

Observe that $T_{b}^{\to }$ includes all basic terms. For the runtime complexity analysis of R, it thus suffices to consider reductions on $T_{b}^{\to }$ only.

### 3.1 Predicative interpretation of terms as sequences

Predicative interpretations separate safe from normal arguments. To this avail, we define the *normalised signature* $F_{n}$ to contain all symbols from F, with the sole difference that the arity of *defined symbols f* with *k* normal arguments is *k* in $F_{n}$. A term *t* is *normalised*, if $t\in T(F_{n})$. Below we retain the separation into constructors, recursive and non-recursive symbols. As a consequence, the rank and recursion depth coincide with respect to both signatures, and also $T(C)\subseteq T(F_{n})$. Terms $T_{b}(F_{n})$ are also called basic, these are obtained from $T_{b}(F)$ by dropping safe arguments.

To formalise sequences of (normalised) terms, we use an auxiliary variadic function symbol ∘. Here variadic means that the arity of ∘ is finite but arbitrary. We always write $\left[t_{1}\;\cdots \;t_{n}\right]$ for $\circ (t_{1},\ldots ,t_{n})$, and if we write $f(t_{1},\ldots ,t_{n})$ then f≠∘. We use a,b,… to denote terms *or* sequences of terms. In contrast, s,t,u,v, possibly followed by subscripts, denote terms which are not sequences. Abusing set-notation, we write $t\in [t_{1}\;\cdots \;t_{n}]$ if $t=t_{i}$ for some i∈{1,…,n}. We lift terms equivalence to sequences by disregarding the order of elements: $\left[s_{1}\;\cdots \;s_{n}]∼[t_{1}\;\cdots \;t_{n}\right]$ if $s_{i}∼t_{\pi (i)}$ for all i=1,…,n and some permutation *π* on {1,…,n}. We denote by a⌢b the *concatenation* of sequences. To avoid notational overhead we overload concatenation to both terms and sequences. For sequences *a* define lift(a):=a, and for terms *t* define lift(t):=[t]. We set $a⌢b:=[s_{1}\;\cdots \;s_{m}\;t_{1}\;\cdots \;t_{n}]$ where $\mathrm{lift}(a)=[s_{1}\;\cdots \;s_{m}]$ and $\mathrm{lift}(b)=[t_{1}\;\cdots \;t_{n}]$.

Definition 3 We define the *predicative interpretation* S, mapping terms $t\in T_{b}^{\to }$ to sequences of normalised terms as follows:

$$S(t):=\left\{ \begin{array}{cc} \left[\;\right] & \text{if}\;t\;\text{is a value,}\\ \left[f(u_{1},\ldots ,u_{k})]⌢S(t_{k+1})⌢\cdots ⌢S(t_{k+l}\right) & \text{otherwise, where}\;t=f(u_{1},\ldots ,u_{k}\;;\;t_{k+1},\ldots ,t_{k+1}). \end{array}\right.$$

Note that the predicative interpretation S(t) is a sequence of (normalised) basic terms for any term $t\in T_{b}^{\to }$. To get the reader prepared for the definition of the order ${⋗}_{\ell }$ on sequences as defined below, we exemplify Definition 3 on a predicative recursive TRS.

Example 6 Consider following predicative recursive TRS $R_{f}$ where

1: f(0;y)→y2: f(s(x);y)→g(x;f(x;y)).

Consider a substitution σ:V→T(C). The embedding $S(l\sigma ){⋗}_{\ell }S(r\sigma )$ of root steps ($l\to r\in R_{f}$) results in the following order constraints.

$$\begin{array}{cccccccc} S(f(0\;;\;y\sigma )) & = & \left[f(0)\right] & {⋗}_{\ell } & \left[\;\right] & = & S(y\sigma ) & \text{by rule}\;\text{1},\\ S(f(s(x\sigma )\;;\;y\sigma )) & = & \left[f(s(x\sigma ))\right] & {⋗}_{\ell } & g(x\sigma )⌢f(x\sigma ) & = & S(g(x\sigma \;;\;f(x\sigma \;;\;y\sigma ))) & \text{by rule}\;\text{2}. \end{array}$$

Kindly observe that in the first line we employed S(yσ)=[] because *yσ* is a value. In the second line we tacitly employed the overloading of concatenation:

S(g(xσ;f(xσ;yσ)))=[g(xσ)]⌢S(f(xσ;yσ))=[g(xσ)]⌢[f(xσ)]⌢[]=g(xσ)⌢f(xσ).

Consider now a rewrite step $s\to_{R}t$ below the root for $s\in T_{b}^{\to }$. As $s\in T_{b}^{\to }$, without loss of generality the rewrite step happens below a safe argument position. Hence

$$s=h(\overset{\to }{v}\;;\;s_{1},\ldots ,s_{i},\ldots ,s_{l})\to_{R}h(\overset{\to }{v}\;;\;s_{1},\ldots ,t_{i},\ldots ,s_{l})=t$$

for some values $\overset{\to }{v}$, terms $s_{1},\ldots ,s_{l}$ and $s_{i}\to_{R}t_{i}$. To embed such rewrite steps we have to prove

$$[h(\overset{\to }{v})]⌢S(s_{1})⌢\cdots ⌢S(s_{i})⌢\cdots ⌢S(s_{l}){⋗}_{\ell }[h(\overset{\to }{v})]⌢S(s_{1})⌢\cdots ⌢S(t_{i})⌢\cdots ⌢S(s_{l}).$$

We emphasise that for a root step $l\sigma \to_{R}r\sigma$ of a predicative recursive TRS R, the length of S(rσ) does not depend on *σ*, since images of *σ* are removed by the predicative interpretation. As a consequence, each step in an R-derivation on $T_{b}^{\to }$ increases the length of predicative interpretations by a constant (depending on R) only. Below, we bind this constant by the maximal size of a right-hand side in R.

### 3.2 Small polynomial path order on sequences

We arrive at the definition of the order ${⋗}_{\ell }$ on sequences. This order is used to orient images of the predicative interpretation S. The parameter ℓ∈N in ${⋗}_{\ell }$ controls the width of terms and sequences, and is crucial for the analysis of the length of ${⋗}_{\ell }$-descending sequences carried out below.

Definition 4 Let ⩾ denote a precedence. For all ℓ⩾1 we define ${⋗}_{\ell }$ on terms and sequences of terms inductively such that:

1. $f(s_{1},\ldots ,s_{n}){⋗}_{\ell }g(t_{1},\ldots ,t_{m})$ if f∈D, f>g and the following conditions hold:
  – $f(s_{1},\ldots ,s_{n})▷{/}_{∼}t_{j}$ for all j=1,…,m;
  – m⩽ℓ.
2. $f(s_{1},\ldots ,s_{n}){⋗}_{\ell }g(t_{1},\ldots ,t_{n})$ if $f\in D_{\mathrm{rec}}$, f∼g and for some permutation *π* on {1,…,n}:
  – $\langle s_{1},\ldots ,s_{n}\rangle ▷{/}_{∼}\langle t_{\pi (1)},\ldots ,t_{\pi (n)}\rangle$.
3. $f(s_{1},\ldots ,s_{n}){⋗}_{\ell }[t_{1}\;\cdots \;t_{m}]$ if the following conditions hold:
  – $f(s_{1},\ldots ,s_{n}){⋗}_{\ell }t_{j}$ for all j=1,…,m;
  – at most one element $t_{j_{0}}$ ($j_{0}\in \{1,\ldots ,m\}$) contains a symbols *g* with f∼g;
  – m⩽ℓ.
4. $\left[s_{1}\;\cdots \;s_{n}]{⋗}_{\ell }[t_{1}\;\cdots \;t_{m}\right]$ if there exists terms *or* sequences $b_{i}$ (i=1,…,n) such that:
  – $[t_{1}\;\cdots \;t_{m}]∼b_{1}⌢\cdots ⌢b_{n}$;
  – $\langle \langle s_{1},\ldots ,s_{n}\rangle \rangle {⋗}_{\ell }\langle \langle b_{1},\ldots ,b_{n}\rangle \rangle$.

We denote by  that either a∼b or $a{⋗}_{\ell }b$ holds. We use $▷{/}_{∼}$ and ${⋗}_{\ell }$ also for their extension to products: $\langle s_{1},\ldots ,s_{n}\rangle ▷{/}_{∼}\langle t_{i},\ldots ,t_{n}\rangle$ if $s_{i}⊵{/}_{∼}t_{i}$ for all i=1,…,n, and $s_{i_{0}}▷{/}_{∼}t_{i_{0}}$ for at least one $i_{0}\in \{1,\ldots ,n\}$; likewise $\langle s_{1},\ldots ,s_{n}\rangle {⋗}_{\ell }\langle t_{i},\ldots ,t_{n}\rangle$ if  for all i=1,…,n, and $s_{i_{0}}{⋗}_{\ell }t_{i_{0}}$ for at least one $i_{0}\in \{1,\ldots ,n\}$.

We point out that ${⋗}_{\ell }$ misses the case: $f(s_{1},\ldots ,s_{n}){⋗}_{\ell }t$ if  for some argument $s_{i}$. Since predicative interpretations remove values, the clause is not needed, compare the embedding of rule 1 given in Example 6. This case would invalidate the central Lemma 7 given below, which estimates the length of ${⋗}_{\ell }$ descending sequences. Observe that on constructor based left-hand sides, the order constraints imposed by ${⋗}_{\ell }^{\langle 1\rangle }$ and ${⋗}_{\ell }^{\langle 2\rangle }$ translate to the order constraints imposed by ${>}_{{\mathrm{spop}}^{⁎}}^{\langle 2\rangle }$ and ${>}_{{\mathrm{spop}}^{⁎}}^{\langle 3\rangle }$ on *normal* arguments. The clauses ${⋗}_{\ell }^{\langle 3\rangle }$ and ${⋗}_{\ell }^{\langle 4\rangle }$ extend the order from terms to sequences. Noteworthy the second clause in ${⋗}_{\ell }^{\langle 3\rangle }$ reflects that we do not allow multiple recursive calls, compare ${>}_{{\mathrm{spop}}^{⁎}}^{\langle 2\rangle }$ and the definition of the predicative interpretation. We exercise Definition 4 on the constraints obtained in Example 6.

Example 7 **Example 6 continued**

We show that the order constraints drawn in Example 6 can be resolved for ℓ=2. Let σ:V→T(C) be a substitution. Consider first the root step $f(0\;;\;y\sigma )\to_{R}y\sigma$ due to rule 1. Exploiting the shape of *σ*, we have $S(f(0\;;\;y\sigma ))=[f(0)]{⋗}_{\ell }^{\langle 4\rangle }[\;]=S(y\sigma )$. For the root step $f(s(x\sigma )\;;\;y\sigma )\to_{R}g(x\sigma \;;\;f(x\sigma \;;\;y\sigma ))$ caused by rule 2 we have

$$\begin{array}{ccccc} \text{1}: & s(x\sigma ) & ▷{/}_{∼} & x\sigma & \\ \text{2}: & f(s(x\sigma )) & ▷{/}_{∼} & x\sigma & \text{by 1,}\\ \text{3}: & f(s(x\sigma )) & {⋗}_{2}^{\langle 1\rangle } & g(x\sigma ) & \text{if}\;f>g,\;\text{using 2},\\ \text{4}: & f(s(x\sigma )) & {⋗}_{2}^{\langle 2\rangle } & f(x\sigma ) & \text{by 1,}\\ \text{5}: & f(s(x\sigma )) & {⋗}_{2}^{\langle 3\rangle } & g(x\sigma )⌢f(x\sigma ) & \text{using 3 and 4,}\\ \text{6}: & S(f(s(x\sigma )\;;\;y\sigma ))=[f(s(x\sigma ))] & {⋗}_{2}^{\langle 4\rangle } & g(x\sigma )⌢f(x\sigma )=g(x\sigma \;;\;f(x\sigma \;;\;y\sigma )) & \text{using 5.} \end{array}$$

Note that g(xσ)⌢f(xσ)=[g(xσ)f(xσ)] and thus ℓ=2 is needed in the proof step 5.

The next lemma collects frequently used properties of ${⋗}_{\ell }$.

Lemma 2 *For all* ℓ⩾1 *we have:*

– ${⋗}_{\ell }\subseteq {⋗}_{\ell +1}$*,*
– $∼\cdot {⋗}_{\ell }\cdot ∼\subseteq {⋗}_{\ell }$*, and*
– $a{⋗}_{\ell }b$ *implies* $a⌢c{⋗}_{\ell }b⌢c$*.*

Proof All but the third property follow directly from definition. Suppose $a{⋗}_{\ell }b$ holds, and let $\mathrm{lift}(c)=[r_{1}\;\cdots \;r_{l}]$. We show $a⌢c{⋗}_{\ell }b⌢c$. First suppose $a=f(s_{1},\ldots ,s_{n})$. Then we conclude

$$a⌢c=[f(s_{1},\ldots ,s_{n})\;r_{1}\;\cdots \;r_{l}]{⋗}_{\ell }^{\langle 4\rangle }b⌢r_{1}⌢\cdots ⌢r_{l}=b⌢c$$

employing the assumption $a{⋗}_{\ell }b$ and $r_{i}∼r_{i}$ for all i=1,…,l. Otherwise $a=[s_{1}\;\cdots \;s_{n}]$, hence $a{⋗}_{\ell }^{\langle 4\rangle }b$ by assumption. Then $b∼b_{1}⌢\cdots ⌢b_{n}$ with  for all i=1,…,n, where at least one orientation is strict. From this and again using $r_{i}∼r_{i}$ (i=1,…,l) we conclude

$$a⌢c=[s_{1}\;\cdots \;s_{n}\;r_{1}\;\cdots \;r_{l}]{⋗}_{\ell }^{\langle 4\rangle }b_{1}⌢\cdots ⌢b_{n}⌢r_{1}⌢\cdots ⌢r_{l}=b⌢c.$$

 □

We emphasise that as a consequence of Lemma 2 we have that $c_{1}⌢a⌢c_{2}{⋗}_{\ell }c_{1}⌢b⌢c_{2}$ holds whenever $a{⋗}_{\ell }b$ holds. The order constraints on sequences are defined so that sequences purely act as containers. More precise, every ${⋗}_{\ell }$-descending sequence starting from $\left[s_{1}\;\cdots \;s_{n}\right]$ can be seen as a combination of possibly interleaved, but otherwise independent ${⋗}_{\ell }$-descending sequences starting from the elements $s_{i}$ (i=1,…,n).

### 3.3 Predicative embedding

We now close the diagram outlined in Fig. 2 on page 9, that is we prove the predicative embedding exemplified in Example 7 on the TRS $R_{f}$. As a preparatory step, we consider root steps $l\sigma \to_{R}r\sigma$ first. The complete embedding is then established in Lemma 4.

Lemma 3 *Consider a rewrite rule* l→r∈R*. Let* σ:V→T(C) *be a substitution. If* $l{>}_{{\mathrm{spop}}^{⁎}}r$ *holds then* $S(l\sigma ){⋗}_{\left|r\right|}S(r\sigma )$*.*

Proof Let $l=f(l_{1},\ldots ,l_{m};l_{m+1},\ldots ,t_{m+n})$. We first show

(⁎) $$l{>}_{{\mathrm{spop}}^{⁎}}r\;⟹\;f(l_{1}\sigma ,\ldots ,l_{m}\sigma ){⋗}_{\left|r\right|}S(t)\;\text{for all}\;t\in S(r\sigma ),$$

by induction on |r|. The non-trivial case is when *rσ* is not a value, otherwise S(rσ)=[]. Suppose thus $r=g(r_{1},\ldots ,r_{m^{\prime }};r_{m^{\prime }+1},\ldots ,r_{m^{\prime }+n^{\prime }})$ where *r* is not a value. By definition

$$S(r\sigma )=[g(r_{1}\sigma ,\ldots ,r_{m^{\prime }}\sigma )]⌢S(r_{m^{\prime }+1}\sigma )⌢\cdots ⌢S(r_{m^{\prime }+n^{\prime }}\sigma ).$$

First consider the element $g(r_{1}\sigma ,\ldots ,r_{m^{\prime }}\sigma )\in S(r\sigma )$. We either have $l{>}_{{\mathrm{spop}}^{⁎}}^{\langle 2\rangle }r$ or $l{>}_{{\mathrm{spop}}^{⁎}}^{\langle 3\rangle }r$ by the assumption that *r* is not a value. In the case $l{>}_{{\mathrm{spop}}^{⁎}}^{\langle 2\rangle }r$, we have f>g and $l{▷}^{\text{n}}{/}_{∼}r_{j}$ for all normal arguments $r_{j}$ $\left(j=1,\ldots ,m^{\prime }\right)$. The latter reveals that the instances $r_{j}\sigma$ are equivalent to proper subterms of the left-hand side $f(l_{1}\sigma ,\ldots ,l_{m}\sigma )$. Using this and that trivially $m^{\prime }⩽|r|$ holds we conclude $f(l_{1}\sigma ,\ldots ,l_{m}\sigma ){⋗}_{\left|r\right|}^{\langle 1\rangle }g(r_{1}\sigma ,\ldots ,r_{m^{\prime }}\sigma )$. In the remaining case $l{>}_{{\mathrm{spop}}^{⁎}}^{\langle 3\rangle }r$, we have $m^{\prime }=m$, f∼g where $f,g\in D_{\mathrm{rec}}$ and moreover $\langle l_{1},\ldots ,l_{m}\rangle {>}_{{\mathrm{spop}}^{⁎}}\langle r_{\pi (1)},\ldots ,r_{\pi (m)}\rangle$ for some permutation *π*. By reasoning as above we see $\langle l_{1}\sigma ,\ldots ,l_{m}\sigma \rangle ▷{/}_{∼}\langle r_{\pi (1)}\sigma ,\ldots ,r_{\pi (m)}\sigma \rangle$ and conclude $f(l_{1}\sigma ,\ldots ,l_{m}\sigma ){⋗}_{\left|r\right|}^{\langle 2\rangle }g(r_{1}\sigma ,\ldots ,r_{m^{\prime }}\sigma )$. Hence overall we obtain $f(l_{1}\sigma ,\ldots ,l_{m}\sigma ){⋗}_{\left|r\right|}g(r_{1}\sigma ,\ldots ,r_{m^{\prime }}\sigma )$.

Now consider the remaining elements t∈S(rσ), where $t\neq g(r_{1}\sigma ,\ldots ,r_{m^{\prime }}\sigma )$. Then each *t* occurs in the interpretation of a safe argument of *rσ*, say $t\in S(r_{j}\sigma )$ for some $j\in \{m^{\prime }+1,\ldots ,m^{\prime }+n^{\prime }\}$. One verifies that, $l{>}_{{\mathrm{spop}}^{⁎}}r_{j}$ holds: if $l{>}_{{\mathrm{spop}}^{⁎}}^{\langle 2\rangle }r$ then by definition, otherwise $l{>}_{{\mathrm{spop}}^{⁎}}^{\langle 3\rangle }r$ holds and we obtain $l{>}_{{\mathrm{spop}}^{⁎}}^{\langle 1\rangle }r_{j}$. By induction hypothesis we have $f(l_{1}\sigma ,\ldots ,l_{m}\sigma ){⋗}_{\left|r_{j}\right|}t$. As ${⋗}_{\left|r_{j}\right|}\subseteq {⋗}_{\left|r\right|}$ we hence obtain $f(l_{1}\sigma ,\ldots ,l_{m}\sigma ){⋗}_{\left|r\right|}t$ for all $t\in S(r_{j}\sigma )$ and safe positions $j\in \{m^{\prime }+1,\ldots ,m^{\prime }+n^{\prime }\}$ of *g*. This concludes (⁎).

We return to the proof of the lemma. A standard induction gives that the length of S(rσ) is bounded by |r|, compare the remark after Example 6. Using that *σ* maps to values, a second induction on $l{>}_{{\mathrm{spop}}^{⁎}}r$ gives that S(rσ) contains at most one (defined) function symbol *g* equivalent to *f*. Summing up, using (⁎) we conclude $f(l_{1}\sigma ,\ldots ,l_{m}\sigma ){⋗}_{\left|r\right|}^{\langle 3\rangle }S(r\sigma )$. Observe that by assumption the direct subterms of *lσ* are values, and thus $S(l\sigma )=[f(l_{1}\sigma ,\ldots ,l_{m}\sigma )]$ by definition. The lemma thus follows by one application of ${⋗}_{\left|r\right|}^{\langle 4\rangle }$.  □

Lemma 4 *Let* R *denote a predicative recursive TRS and let* ℓ:=max⁡{|r||l→r∈R}*. If* $s\in T_{b}^{\to }$ *and* $s\to_{R}t$ *then* $S(s){⋗}_{\ell }S(t)$*.*

Proof Let $s\in T_{b}^{\to }$ and consider a rewrite step $s\to_{R}t$. We prove the lemma by induction on the rewrite position. In the base case we consider a root step $s=l\sigma \to_{R}r\sigma =t$ for some rule l→r∈R. Since R is predicative recursive, $l{>}_{{\mathrm{spop}}^{⁎}}r$ holds. By Lemma 3 we have $S(l\sigma ){⋗}_{\left|r\right|}S(r\sigma )$. Since |r|⩽ℓ the result follows.

For the inductive step, consider a rewrite step below the root. Since $s\in T_{b}^{\to }$ this step is of the form

$$s=f(\overset{\to }{v}\;;\;s_{1},\ldots ,s_{i},\ldots ,s_{n})\to_{R}f(\overset{\to }{v}\;;\;s_{1},\ldots ,t_{i},\ldots ,s_{n})=t,$$

where $s_{i}\to_{R}t_{i}$ for some i∈{1,…,n}. Wlog. we assume *t* is not a value. Using the induction hypothesis $S(s_{i}){⋗}_{\ell }S(t_{i})$ and Lemma 2 we conclude

$$S(s\sigma )=f(\overset{\to }{v})⌢S(s_{1})⌢\cdots ⌢S(s_{i})⌢\cdots ⌢S(s_{n}){⋗}_{\ell }f(\overset{\to }{v})⌢S(s_{1})⌢\cdots ⌢S(t_{i})⌢\cdots ⌢S(s_{n})=S(t\sigma ).$$

 □

### 3.4 Binding the length of ${⋗}_{\ell }$-descending sequences

The following function $G_{\ell }$ relates a term, or sequence of terms, to the length of its longest ${⋗}_{\ell }$-descending sequence.

Definition 5 For all ℓ⩾1, we define $G_{\ell }(a):=\max\{m|a{⋗}_{\ell }a_{1}{⋗}_{\ell }\cdots {⋗}_{\ell }a_{m}\}$.

This function is well-defined, as ${⋗}_{\ell }$ is well-founded. The latter can be seen as ${⋗}_{\ell }$ forms a restriction of the multiset path order [35]. We remark that due to Lemma 2, $G_{\ell }(a)=G_{\ell }(b)$ whenever a∼b. The following lemma confirms that sequences act as containers only.

Lemma 5 *For all sequences* $\left[s_{1}\;\cdots \;s_{n}\right]$*,* $G_{\ell }([s_{1}\;\cdots \;s_{n}])=\sum_{i=1}^{n}G_{\ell }(s_{i})$*.*

Proof Let $a=[s_{1}\;\cdots \;s_{n}]$ be a sequence and observe $G_{\ell }(a_{1}⌢a_{2})⩾G_{\ell }(a_{1})+G_{\ell }(a_{2})$. This is a consequence of Lemma 2. Hence, in particular we obtain: $G_{\ell }(a)=G_{\ell }(s_{1}⌢\cdots ⌢s_{n})⩾\sum_{i=1}^{n}G_{\ell }(s_{i})$.

To complete the proof, we proceed by induction on $G_{\ell }(a)$. The base case $G_{\ell }(a)=0$ follows trivially. For the induction step, we show that $a{⋗}_{\ell }b$ implies $G_{\ell }(b)<\sum_{i=1}^{n}G_{\ell }(s_{i})$. From this, we obtain $G_{\ell }([s_{1}\;\cdots \;s_{n}])⩽\sum_{i=1}^{n}G_{\ell }(s_{i})$, which together with the above observation yields $G_{\ell }([s_{1}\;\cdots \;s_{n}])=\sum_{i=1}^{n}G_{\ell }(s_{i})$. Suppose $a{⋗}_{\ell }b$. Then this is only possible due to ${⋗}_{\ell }^{\langle 4\rangle }$. Hence *b* is equivalent to $b_{1}⌢\cdots ⌢b_{n}$, where  for all i=1,…,n and $s_{i_{0}}{⋗}_{\ell }b_{i_{0}}$ for at least one $i_{0}\in \{1,\ldots ,n\}$. In particular, $G_{\ell }(b_{i})⩽G_{\ell }(s_{i})$ and $G_{\ell }(b_{i_{0}})<G_{\ell }(s_{i_{0}})$. As we have $G_{\ell }(b_{i})⩽G_{\ell }(b)<G_{\ell }(a)$ for all i=1,…,n, induction hypothesis is applicable to *b* and all $b_{i}$ (i∈{1,…,n}). It follows that

$$G_{\ell }(b)=\sum_{t\in b}G_{\ell }(t)=\sum_{i=1}^{n}\sum_{t\in b_{i}}G_{\ell }(t)=\sum_{i=1}^{n}G_{\ell }(b_{i})<\sum_{i=1}^{n}G_{\ell }(s_{i}).$$

 □

We now approach Lemma 7, where we show that $G_{\ell }(f(u_{1},\ldots ,u_{k}))⩽c\cdot {\left(2+m\right)}^{\mathrm{rd}(f)}$ for some constant c∈N and $m=\sum_{i=1}^{k}\mathrm{dp}(u_{i})$. The proof of Lemma 7 is slightly involved, and requires induction on the rank *r* of *f* and side induction on *m*. The constant *c* is defined in terms of c(r,d) for natural numbers r,d∈N:

$$c(r,d):=\left\{ \begin{array}{cc} 1 & \text{if}\;r=1,\;\text{and}\\ c(r-1,d)\cdot \ell^{d+1}+1 & \text{otherwise}. \end{array}\right.$$

Below the argument *r* will be instantiated by the rank, and *d* by the depth of recursion of a function symbol *f*. The next lemma is a technical lemma to ease the presentation of the proof of Lemma 7. The assumptions correspond exactly to the main induction hypothesis (IH) and side induction hypothesis (SIH) of Lemma 7.

Lemma 6 *Consider* $f(u_{1},\ldots ,u_{k}){⋗}_{\ell }g(v_{1},\ldots ,v_{l})$ *and suppose that*

(IH) $$f>g\;⟹\;G_{\ell }(g(v_{1},\ldots ,v_{l}))⩽c(\mathrm{rk}(g),\mathrm{rd}(g))\cdot {\left(2+\sum_{i=1}^{l}\mathrm{dp}(v_{i})\right)}^{\mathrm{rd}(g)},$$

(SIH) $$f∼g,\sum_{i=1}^{l}\mathrm{dp}(v_{i})<\sum_{i=1}^{k}\mathrm{dp}(u_{i})\;⟹\;G_{\ell }(g(v_{1},\ldots ,v_{l}))⩽c(\mathrm{rk}(g),\mathrm{rd}(g))\cdot {\left(2+\sum_{i=1}^{l}\mathrm{dp}(v_{i})\right)}^{\mathrm{rd}(g)}.$$

*Then*

(†) $$f(u_{1},\ldots ,u_{k}){⋗}_{\ell }^{\langle 1\rangle }g(v_{1},\ldots ,v_{l})\;⟹\;G_{\ell }(g(v_{1},\ldots ,v_{l}))⩽c(\mathrm{rk}(f)-1,\mathrm{rd}(f))\cdot \ell^{\mathrm{rd}(f)}\cdot {\left(2+\sum_{i=1}^{k}\mathrm{dp}(u_{i})\right)}^{\mathrm{rd}(g)},$$

(‡) $$f(u_{1},\ldots ,u_{k}){⋗}_{\ell }^{\langle 2\rangle }g(v_{1},\ldots ,v_{l})\;⟹\;G_{\ell }(g(v_{1},\ldots ,v_{l}))⩽c(\mathrm{rk}(f),\mathrm{rd}(f))\cdot {\left(1+\sum_{i=1}^{k}\mathrm{dp}(u_{i})\right)}^{\mathrm{rd}(f)}.$$

Proof First consider the case $f(u_{1},\ldots ,u_{k}){⋗}_{\ell }^{\langle 1\rangle }g(v_{1},\ldots ,v_{l})$. Then f>g and so rk(f)>rk(g) and rd(f)⩾rd(g) hold. From the order constraints on arguments we can derive $\sum_{i=1}^{l}\mathrm{dp}(v_{i})⩽l\cdot \sum_{i=1}^{k}\mathrm{dp}(u_{i})$. Observe that the assumption gives also l⩽ℓ. Summing up, simple arithmetical reasoning gives the implication (†) from (IH). Similar, when $f(u_{1},\ldots ,u_{k}){⋗}_{\ell }^{\langle 2\rangle }g(v_{1},\ldots ,v_{l})$ holds we have rk(f)=rk(g) and rd(f)=rd(g). The order constraints on arguments give $\sum_{i=1}^{l}\mathrm{dp}(v_{i})<\sum_{i=1}^{k}\mathrm{dp}(u_{i})$. From this, the implication (‡) follows directly from (SIH).  □

Lemma 7 *For all* f∈D*,* $G_{\ell }(f(u_{1},\ldots ,u_{k}))\in O({\left(\sum_{i=1}^{k}\mathrm{dp}(u_{i})\right)}^{\mathrm{rd}(f)})$*.*

Proof Let *ℓ* be fixed. To show the theorem, we show

$$f(u_{1},\ldots ,u_{k}){⋗}_{\ell }b\;⟹\;G_{\ell }(b)<c(\mathrm{rk}(f),\mathrm{rd}(f))\cdot {\left(2+\sum_{i=1}^{k}\mathrm{dp}(u_{i})\right)}^{\mathrm{rd}(f)}.$$

In proof we employ induction on rk(f) and side induction on $m:=\sum_{i=1}^{k}\mathrm{dp}(u_{i})$. Abbreviate r:=rk(f) and d:=rd(f). Assume that $f(u_{1},\ldots ,u_{k}){⋗}_{\ell }b$ holds. We prove $G_{\ell }(b)<c(r,d)\cdot {\left(2+m\right)}^{d}$, where we show only the more involved inductive case r>1. The base case r=1 follows by similar reasoning. We analyse two cases.

If $b=[t_{1}\;\cdots \;t_{l}]$ is a sequence, then by assumption $f(v_{1},\ldots ,v_{k}){⋗}_{\ell }^{\langle 3\rangle }b$. Thus l⩽ℓ and $f(v_{1},\ldots ,v_{k}){⋗}_{\ell }t_{j}$, i.e. either $f(v_{1},\ldots ,v_{k}){⋗}_{\ell }^{\langle 1\rangle }t_{j}$ or $f(v_{1},\ldots ,v_{k}){⋗}_{\ell }^{\langle 2\rangle }t_{j}$ holds for all j=1,…,l. Due to the second condition imposed on ${⋗}_{\ell }^{\langle 3\rangle }$, we even have $f(v_{1},\ldots ,v_{k}){⋗}_{\ell }^{\langle 1\rangle }t_{j}$ for all but one $j\neq j_{0}\in \{1,\ldots ,l\}$. Suppose first that *f* is recursive. Then $f(v_{1},\ldots ,v_{k}){⋗}_{\ell }^{\langle 1\rangle }t_{j}$ implies $d>\mathrm{rd}(\mathrm{rt}(t_{j}))⩾0$. Using induction and side induction hypothesis to satisfy the assumptions of Lemma 6, we obtain

$$\begin{array}{cc} G_{\ell }(t_{j})⩽c(r-1,d)\cdot \ell^{d}\cdot {\left(2+m\right)}^{d-1} & \;(\text{for all}j\neq j_{0})\\ G_{\ell }(t_{j_{0}})⩽\max\{c(r-1,d)\cdot \ell^{d}\cdot {\left(2+m\right)}^{d-1},c(r,d)\cdot {\left(1+m\right)}^{d}\}. & \end{array}$$

Here the second inequality is obtained by combining the conclusions of the two implications provided by Lemma 6. We conclude the case as follows.

$$\begin{array}{cccc} G_{\ell }(b) & = & \sum_{j=1}^{l}G_{\ell }(t_{j}) & \text{by Lemma 5,}\\ & ⩽ & c(r,d)\cdot {\left(1+m\right)}^{d}+l\cdot (c(r-1,d)\cdot \ell^{d}\cdot {\left(2+m\right)}^{d-1}) & \text{above consequences of Lemma 6,}\\ & < & c(r,d)\cdot {\left(1+m\right)}^{d}+c(r,d)\cdot {\left(2+m\right)}^{d-1} & \text{using}\;l⩽\ell \;\text{and unfolding}\;c(r,d),\\ & ⩽ & c(r,d)\cdot {\left(2+m\right)}^{d}. & \end{array}$$

Suppose now that *f* is not recursive. Then also $f(v_{1},\ldots ,v_{k}){⋗}_{\ell }^{\langle 1\rangle }t_{j_{0}}$. Employing that $f>\mathrm{rt}(t_{j})$ implies $d⩾\mathrm{rd}(\mathrm{rt}(t_{j}))$, using Lemma 5 and Lemma 6 we see

$$G_{\ell }(b)=\sum_{j=1}^{l}G_{\ell }(t_{j})⩽l\cdot (c(r-1,d)\cdot \ell^{d}\cdot {\left(2+m\right)}^{d})<c(r,d)\cdot {\left(2+m\right)}^{d}.$$

This finishes the cases when *b* is a sequence.

Finally, when $b=g(t_{1},\ldots ,t_{l})$ is a term we conclude directly by Lemma 6, using $c(r-1,d)\cdot \ell^{d}<c(r,d)$ similar to above.  □

Putting things together, we arrive at the proof of our first theorem.

Proof of Theorem 1 Let R denote a predicative recursive TRS. We prove the existence of a constant c∈N such that for all values $\overset{\to }{u},\overset{\to }{v}$ the derivation height of $f(\overset{\to }{u}\;;\;\overset{\to }{v})$ is bounded by $c\cdot n^{\mathrm{rd}(f)}$, where *n* is the sum of the depths of normal arguments $\overset{\to }{u}$.

Consider a derivation $f(\overset{\to }{u}\;;\;\overset{\to }{v})\to_{R}t_{1}\to_{R}\cdots \to_{R}t_{n}$. Let i∈{0,…,n−1}. By Lemma 1 it follows that $t_{i}\in T_{b}^{\to }$, and consequently $S(t_{i}){⋗}_{\ell }S(t_{i+1})$ due to Lemma 4. So in particular the length *n* is bounded by the length of ${⋗}_{\ell }$ descending sequences starting from $S(f(\overset{\to }{u}\;;\;\overset{\to }{v}))=[f(\overset{\to }{u})]$. By Lemma 5, $G_{\ell }([f(\overset{\to }{u})])=G_{\ell }(f(\overset{\to }{u}))$. Thus Lemma 7 gives the constant c∈N as desired.  □

## 4 Parameter substitution

Bellantoni already observed that his definition of FP is closed under safe recursion on notation with *parameter substitution*. Here a function *f* is defined from functions $g,h_{0},h_{1}$ and $\overset{\to }{p}$ by

(${\mathrm{SRN}}_{\mathrm{PS}}$) $$\begin{array}{ccc} f(\epsilon ,\overset{\to }{x}\;;\;\overset{\to }{y}) & = & g(\overset{\to }{x}\;;\;\overset{\to }{y})\\ f(zi,\overset{\to }{x}\;;\;\overset{\to }{y}) & = & h_{i}(z,\overset{\to }{x}\;;\;\overset{\to }{y},f(z,\overset{\to }{x}\;;\;\overset{\to }{p}(z,\overset{\to }{x}\;;\;\overset{\to }{y})))\;(i=0,1). \end{array}$$

We introduce the *small polynomial path order with parameter substitution* (${\mathrm{sPOP}}_{\mathrm{SP}}^{⁎}$ for short), where clause ${>}_{{\mathrm{spop}}^{⁎}}^{\langle 3\rangle }$ is extended to account for the schema $\left({\mathrm{SRN}}_{\mathrm{PS}}\right)$. Theorem 1 remains valid under this extension.

Definition 6 Let *s* and *t* be terms such that $s=f(s_{1},\ldots ,s_{k};s_{k+1},\ldots ,s_{k+l})$. Then $s{>}_{{\mathrm{spop}}_{\mathrm{ps}}^{⁎}}t$ if one of the following alternatives holds.

1. $s_{i}{⩾}_{{\mathrm{spop}}_{\mathrm{ps}}^{⁎}}t$ for some argument $s_{i}$ of *s* (i∈{1,…,k+l}).
2. f∈D, $t=g(t_{1},\ldots ,t_{m};t_{m+1},\ldots ,t_{m+n})$ where f>g and the following holds:
  – $s{▷}^{\text{n}}{/}_{∼}t_{j}$ for all normal arguments $t_{1},\ldots ,t_{m}$;
  – $s{>}_{{\mathrm{spop}}_{\mathrm{ps}}^{⁎}}t_{j}$ for all safe arguments $t_{m+1},\ldots ,t_{m+n}$;
  – *t* contains at most one (recursive) function symbols *g* with f∼g.
3. $f\in D_{\mathrm{rec}}$, $t=g(t_{1},\ldots ,t_{k};t_{k+1},\ldots ,t_{k+l})$ where f∼g and the following holds:
  – $\langle s_{1},\ldots ,s_{k}\rangle {>}_{{\mathrm{spop}}_{\mathrm{ps}}^{⁎}}\langle t_{\pi (1)},\ldots ,t_{\pi (k)}\rangle$ for some permutation *π* on {1,…,k};
  – $s{>}_{{\mathrm{spop}}_{\mathrm{ps}}^{⁎}}t_{j}$ for all safe arguments $t_{j}$;
  – arguments $t_{1},\ldots ,t_{k+l}$ contain no (recursive) symbols *g* with f∼g.

Here $s{⩾}_{{\mathrm{spop}}_{\mathrm{ps}}^{⁎}}t$ denotes that either $s\overset{s}{∼}t$ or $s{>}_{{\mathrm{spop}}_{\mathrm{ps}}^{⁎}}t$. In the last clause, we use ${>}_{{\mathrm{spop}}_{\mathrm{ps}}^{⁎}}$ also for the product extension of ${>}_{{\mathrm{spop}}_{\mathrm{ps}}^{⁎}}$ (modulo permutation).

We adapt the notion of predicative recursive TRS of degree *d* to ${\text{sPOP}}_{\mathrm{PS}}^{⁎}$  in the obvious way. Note that ${>}_{{\mathrm{spop}}^{⁎}}\subseteq {>}_{{\mathrm{spop}}_{\mathrm{ps}}^{⁎}}$ does *not* hold in general, due to the third constraint put onto ${>}_{{\mathrm{spop}}_{\mathrm{ps}}^{⁎}}^{\langle 3\rangle }$. Still, the above order extends the analytic strength of small polynomial path orders.

Lemma 8 *If a TRS* R *is compatible with* ${>}_{{\mathrm{spop}}^{⁎}}$ *then* R *is also compatible with* ${>}_{{\mathrm{spop}}_{\mathrm{ps}}^{⁎}}$ *using the same precedence and separation of argument positions.*

Proof Consider the orientation $l{>}_{{\mathrm{spop}}^{⁎}}r$ of a rule l→r∈R. To prove the lemma, we show that $l{>}_{{\mathrm{spop}}_{\mathrm{ps}}^{⁎}}r$ holds by replacing every application of ${>}_{{\mathrm{spop}}^{⁎}}^{\langle i\rangle }$ by ${>}_{{\mathrm{spop}}_{\mathrm{ps}}^{⁎}}^{\langle i\rangle }$. We prove this claim by induction on ${>}_{{\mathrm{spop}}^{⁎}}$. We consider the only non-trivial case where $s{>}_{{\mathrm{spop}}^{⁎}}^{\langle 3\rangle }t$ appears in the proof of $l{>}_{{\mathrm{spop}}_{\mathrm{ps}}^{⁎}}r$. Compare the case ${>}_{{\mathrm{spop}}^{⁎}}^{\langle 3\rangle }$ with the new case ${>}_{{\mathrm{spop}}_{\mathrm{ps}}^{⁎}}^{\langle 3\rangle }$. Using the induction hypothesis, the order constraints on normal arguments are immediately satisfied. Now fix a safe argument $t_{j}$ of *t*. From $s{>}_{{\mathrm{spop}}^{⁎}}^{\langle 3\rangle }t$ we obtain a safe argument $s_{i}$ of *s* with $s_{i}{>}_{{\mathrm{spop}}^{⁎}}t_{j}$. Hence $s_{i}{>}_{{\mathrm{spop}}_{\mathrm{ps}}^{⁎}}t_{j}$ holds by induction hypothesis. Thus $s{>}_{{\mathrm{spop}}_{\mathrm{ps}}^{⁎}}^{\langle 1\rangle }t_{j}$ holds as desired. Observe that the safe arguments $s_{i}$ of *s* are proper subterms of the left-hand side *l*, hence the terms $s_{i}$ contain no defined symbols. Since ${>}_{{\mathrm{spop}}^{⁎}}$ collapses to the subterm relation on constructor terms, it follows that the safe argument $t_{j}$ of *t* are constructor terms too. From this we see that the final constraint of ${>}_{{\mathrm{spop}}_{\mathrm{ps}}^{⁎}}^{\langle 3\rangle }$ is satisfied.  □

Parameter substitution extends the analytic power of ${\text{sPOP}}^{⁎}$ significantly. Noteworthy, ${\text{sPOP}}_{\mathrm{PS}}^{⁎}$  can deal with *tail-recursive* rewrite systems.

Example 8 The TRS $R_{\mathrm{rev}}$ consisting of the three rules

$$\mathrm{rev}(xs\;;\;)\to {\mathrm{rev}}_{\mathrm{tl}}(xs\;;\;\mathrm{nil})\;{\mathrm{rev}}_{\mathrm{tl}}(\mathrm{nil}\;;\;ys)\to ys\;{\mathrm{rev}}_{\mathrm{tl}}(\mathrm{cons}(\;;\;x,xs)\;;\;ys)\to {\mathrm{rev}}_{\mathrm{tl}}(xs\;;\;\mathrm{cons}(\;;\;x,ys)),$$

which reverses lists formed from the constructors nil and cons. Define the separation of argument positions as indicated in the rules. The underlying precedence is given as $\mathrm{rev}>{\mathrm{rev}}_{\mathrm{tl}}>\mathrm{cons}$. Since ${\mathrm{rev}}_{\mathrm{tl}}$ is the only recursive symbol, the degree of recursion of $R_{\mathrm{rev}}$ is one.

Notice that orientation of the final rule with the induced ${\text{sPOP}}^{⁎}$ reduces to the unsatisfiable constraint $ys{>}_{{\mathrm{spop}}^{⁎}}\mathrm{cons}(\;;\;x,ys)$. In contrast, orientation with the induced ${\text{POP}}_{\mathrm{PS}}^{⁎}$  reduces to the constraint ${\mathrm{rev}}_{\mathrm{tl}}(\mathrm{cons}(\;;\;x,xs)\;;\;ys){>}_{{\mathrm{spop}}_{\mathrm{ps}}^{⁎}}\mathrm{cons}(\;;\;x,ys)$, which can be resolved by one application of ${>}_{{\mathrm{spop}}_{\mathrm{ps}}^{⁎}}^{\langle 2\rangle }$ followed by three applications of ${>}_{{\mathrm{spop}}_{\mathrm{ps}}^{⁎}}^{\langle 1\rangle }$.

As a consequence of the next theorem, the runtime of $R_{\mathrm{rev}}$ is inferred to be linear.

Theorem 2 *Let* R *be a predicative recursive TRS of degree d (with respect to* Definition 6*). Then the derivation height of any basic term* $f(\overset{\to }{u}\;;\;\overset{\to }{v})$ *is bounded by a polynomial of degree* rd(f) *in the sum of the depths of normal arguments* $\overset{\to }{u}$*. In particular, the runtime complexity function* ${\mathrm{rc}}_{R}$ *is bounded by a polynomial of degree d.*

Proof We observe that under the new definition all proofs, in particular the predicative embedding shown in Section 3.3, go through unchanged.  □

## 5 Predicative recursive rewrite systems compute all polytime functions

In this section we show that ${\text{sPOP}}^{⁎}$ is complete for FP. Indeed, we can even show a stronger result. Let *f* be a function from $B_{\mathrm{wsc}}$ that makes only use of *d* nestings of safe recursion on notation, then there exists a predicative recursive TRS $R_{f}$ of degree *d* that computes the function *f*.

By definition $B_{\mathrm{wsc}}\subseteq B$ for Bellantoni and Cooks predicative recursive characterisation B of FP given in [1]. Concerning the converse inclusion, the following theorem states that the class $B_{\mathrm{wsc}}$ is large enough to capture *all* the polytime computable functions.

Theorem 3 *Every polynomial time computable function belongs to* $B_{\mathrm{wsc}}$*.*

One can show this fact by following the proof of Theorem 3.7 in [28], where the unary variant of $B_{\mathrm{wsc}}$ is defined and the inclusion corresponding to Theorem 3 is shown. The completeness of ${\text{sPOP}}^{⁎}$ for the polytime computable functions is an immediate consequence of Theorem 3 and the following result.

Theorem 4 *For any* $B_{\mathrm{wsc}}$*-function f there exists a predicative recursive TRS* R *computing f and of degree d, where d equals the maximal number of nested application of* (**SSRN**) *in the definition of f.*

Proof Let *f* be a function coming from $B_{\mathrm{wsc}}$. A witnessing TRS R is obtained via a term rewriting characterisation of the class $B_{\mathrm{wsc}}$ depicted in Fig. 1 on page 5. The term rewriting characterisation expresses the definition of $B_{\mathrm{wsc}}$ as an *infinite* TRS $R_{B_{\mathrm{wsc}}}$. We define a one-to-one correspondence between functions from $B_{\mathrm{wsc}}$ and the set of function symbols for $R_{B_{\mathrm{wsc}}}$ as follows. Constructor symbols *ϵ*, $s_{0}$ and $s_{1}$ are used to denote binary words. The function symbols $S_{i}$, P, $I_{j}^{k,l}$, C and $O^{k,l}$ correspond respectively to the initial functions $S_{i}$, *P*, $I_{j}^{k,l}$, *C* and $O^{k,l}$ of $B_{\mathrm{wsc}}$. The symbol $\mathrm{SUB}[h,i_{1},\ldots ,i_{n},\overset{\to }{g}]$ is used to denote the function obtained by composing functions *h* and $\overset{\to }{g}$ according to the schema of (**WSC**). Finally, the function symbol $\mathrm{SRN}[g,h_{0},h_{1}]$ corresponds to the function defined by safe recursion on notation from *g*, $h_{0}$ and $h_{1}$ in accordance to the schema (**SRN**). With this correspondence, $R_{B_{\mathrm{wsc}}}$ is obtained by orienting the equations in Fig. 1 from left to right.

By induction according to the definition of *f* in $B_{\mathrm{wsc}}$ we show the existence of a TRS $R_{f}$ and a precedence ${⩾}_{f}$ such that:

1. $R_{f}$ is a finite restriction of $R_{B_{\mathrm{wsc}}}$,
2. $R_{f}$ contains the rule(s) defining the function symbol f corresponding to *f*,
3. $R_{f}$ is compatible with ${>}_{{\mathrm{spop}}^{⁎}}$ induced by ${⩾}_{f}$,
4. f is maximal in the precedence ${⩾}_{f}$ underlying $R_{f}$, and
5. the depth of recursion rd(f) equals the maximal number of nested application of (**SRN**) in the definition of *f* in $B_{\mathrm{wsc}}$.

It can be seen from conditions (1), (3) and (5) that the theorem is witnessed by $R_{f}$. To exemplify the construction we consider the step case that *f* is defined from some functions $g,h_{0},h_{1}\in B_{\mathrm{wsc}}$ by the schema (**SRN**). By induction hypothesis we can find witnessing TRSs $R_{g},R_{h_{0}},R_{h_{1}}$ with underlying precedences ${⩾}_{g},{⩾}_{h_{0}},{⩾}_{h_{1}}$ respectively for $g,h_{0},h_{1}$. Extend the set of function symbols by a new recursive symbol $f:=\mathrm{SRN}[g,h_{0},h_{1}]$. Let $R_{f}$ be the TRS consisting of $R_{g}$, $R_{h_{0}}$, $R_{h_{1}}$ and the following three rules:

$$f(\epsilon ,\overset{\to }{x}\;;\;\overset{\to }{y})\to g(\overset{\to }{x}\;;\;\overset{\to }{y})\;f(s_{i}(;x),\overset{\to }{x}\;;\;\overset{\to }{y})\to h_{i}(z,\overset{\to }{x}\;;\;\overset{\to }{y},f(z,\overset{\to }{x}\;;\;\overset{\to }{y}))\;(i=0,1).$$

It is not difficult to see that the precedence ${⩾}_{f}$ of $R_{f}$ extends the precedences ${⩾}_{g}$, ${⩾}_{h_{0}}$ and ${⩾}_{h_{1}}$ by f∼f and $f>g^{\prime }$ for $g^{\prime }\in \{g,h_{0},h_{1}\}$.

Let ${>}_{{\mathrm{spop}}^{⁎}}$ be the ${\text{sPOP}}^{⁎}$ induced by ${⩾}_{f}$. Then it is easy to check that $R_{f}$ enjoys conditions (1) and (2). In order to show condition (3), it suffices to orient the three new rules by ${>}_{{\mathrm{spop}}^{⁎}}$. For the first rule, $f(\epsilon ,\overset{\to }{x};\overset{\to }{y}){>}_{{\mathrm{spop}}^{⁎}}^{\langle 2\rangle }g(\overset{\to }{x};\overset{\to }{y})$ holds by the definition of ${⩾}_{f}$. For the remaining two rules we only orient the case i=0. Since f is a recursive symbol and $s_{0}(;z){>}_{{\mathrm{spop}}^{⁎}}^{\langle 1\rangle }z$ holds, $f(s_{0}(;z),\overset{\to }{x}\;;\;\overset{\to }{y}){>}_{{\mathrm{spop}}^{⁎}}^{\langle 3\rangle }f(z,\overset{\to }{x}\;;\;\overset{\to }{y})$ holds. This together with the definition of the precedence ${⩾}_{f}$ allows us to conclude $f(s_{0}(;z),\overset{\to }{x}\;;\;\overset{\to }{y}){>}_{{\mathrm{spop}}^{⁎}}^{\langle 2\rangle }h_{0}(z,\overset{\to }{x}\;;\;\overset{\to }{y},f(z,\overset{\to }{x}\;;\;\overset{\to }{y}))$.

Consider condition (4). For each function $g^{\prime }\in \{g,h_{0},h_{1}\}$ from $B_{\mathrm{wsc}}$, the corresponding function symbol $g^{\prime }$ is maximal in the precedence ${⩾}_{g^{\prime }}$ by induction hypothesis for $g^{\prime }$. Hence by the definition of ${⩾}_{f}$, f is maximal in ${⩾}_{f}$.

It remains to show condition (5). Notice that $\mathrm{rd}(f)=1+\max\{\mathrm{rd}(g),\mathrm{rd}(h_{0}),\mathrm{rd}(h_{1})\}$, since f is a recursive symbol. Without loss of generality let us suppose $\mathrm{rd}(g)=\max\{\mathrm{rd}(g),\mathrm{rd}(h_{0}),\mathrm{rd}(h_{1})\}$. Then by induction hypothesis for *g*, rd(g) equals the maximal number of nested application of (**SRN**) in the definition of *g* in $B_{\mathrm{wsc}}$. Hence rd(f)=1+rd(g) equals the one in the definition of *f* in $B_{\mathrm{wsc}}$.  □

We obtain that predicative recursive TRSs give a *sound* and *complete* characterisation of the polytime computable functions.

Theorem 5 *The following classes of functions are equivalent:*

1. *The class of functions computed by predicative recursive TRSs.*
2. *The class of functions computed by predicative recursive TRSs allowing parameter substitution.*
3. *The class* FP *of functions computable in polynomial time.*

Proof Let ${\mathrm{PR}}_{1}$ and ${\mathrm{PR}}_{2}$ refer to the classes defined in clauses (1) and (2) respectively. We have

$${\mathrm{PR}}_{1}\overset{\left(\text{Def.}\right)}{\subseteq }{\mathrm{PR}}_{2}\overset{\left(\text{Thm.}\;2\right)}{\subseteq }\mathrm{FP}\overset{\left(\text{Thm.}\;3\right)}{\subseteq }B_{\mathrm{wsc}}\overset{\left(\text{Thm.}\;4\right)}{\subseteq }{\mathrm{PR}}_{1}.$$

For the second inclusion we tacitly employed the adequacy theorem.  □

We remark that from our standing restriction on TRSs, orthogonality is essentially used to ensure that semantics of TRSs are well-defined. Orthogonality could be replaced by the less restrictive, although undecidable, notion of confluence.

As a corollary to Theorem 5 we obtain that the class FP, viz $B_{\mathrm{wsc}}$, is closed under parameter substitution.

Corollary 1 *For any functions* $g,h_{0},h_{1},\overset{\to }{p}\in B_{\mathrm{wsc}}$*, there exists a unique polytime computable function f such that* $f(\epsilon ,\overset{\to }{x}\;;\;\overset{\to }{y})=g(\overset{\to }{x}\;;\;\overset{\to }{y})$ *and* $f(zi,\overset{\to }{x}\;;\;\overset{\to }{y})=h_{i}(z,\overset{\to }{x}\;;\;\overset{\to }{y},f(z,\overset{\to }{x}\;;\;\overset{\to }{p}(z,\overset{\to }{x}\;;\;\overset{\to }{y})))$ *for each* i=0,1*.*

## 6 Predicative recursion precisely captures register machine computations

Exploiting the fine-grained control given by the degree of recursion, we now provide an order-theoretic characterisation of $\mathrm{DTIME}(n^{d})$ via *predicative tail-recursive* TRSs.

Definition 7 A TRS R is *tail-recursive* if for every rule $f(\overset{\to }{v})\to r\in R$, if *g* with g∼f occurs in *r* then it occurs at the root position in *r*. The TRS R is *predicative tail-recursive* (of degree *d*), if it is tail-recursive and predicative recursive (of degree *d*), with respect to Definition 6.

For instance, the TRS $R_{\mathrm{rev}}$ from Example 8 is a predicative tail-recursive TRS. The restriction to tail-recursion is unarguably severe. Still, predicative tail-recursive TRSs of degree *d* can compute polynomials $c\cdot n^{d}+e$ (in unary notation) for all c,e∈N.

Example 9 Let $p(n):=c\cdot n^{d}+e$ denote a polynomial with constants c,d,e∈N. The TRS $R_{p}$ is given by the following rules.

$$\begin{array}{cccc} p_{0}(x,y\;;\;z) & \to & s^{c}(\;;\;z)\\ p_{r}(0,y\;;\;z) & \to & z & \text{for}\;r=1,\ldots ,d,\\ p_{r}(s(\;;\;x),y\;;\;z) & \to & p_{r}(x,y\;;\;p_{r-1}(y,y\;;\;z))) & \text{for}\;r=1,\ldots ,d,\\ p(x\;;\;) & \to & p_{d}(x,x\;;\;s^{e}(\;;\;0)) & \end{array}$$

This TRS is tail-recursive, moreover it is predicative recursive with recursive symbols $p_{1},\ldots ,p_{d}$ and precedence $p_{d}>\cdots p_{1}>p_{0}>s∼0$. In total, $R_{p}$ is thus predicative tail-recursive, of degree *d*.

Let $⌜n⌝=s^{n}(\;;\;0)$ denote the denotation of n∈N as value with constructors s and 0. One verifies that for u,v,w∈N, $p_{r}(⌜u⌝,⌜v⌝,⌜w⌝)$ reduces to the value $⌜c\cdot u\cdot v^{r-1}+w⌝$, for r=1,…,d. Thus $〚p〛(⌜n⌝)=⌜c\cdot n\cdot n^{d-1}+e⌝=⌜c\cdot n^{d}+e⌝$ for all n∈N.

### 6.1 Predicative tail-recursive TRSs of degree *d* are complete for $\mathrm{DTIME}(n^{d})$

Fix a register machine *M* that computes a function $f\;:\;W{\left(A\right)}^{k}\to W(A)$ in time $O(n^{d})$. We show that this function is computable by a predicative tail-recursive TRS of degree *d*. Let $C_{A}$ denote the set of constructors that contains a symbol *ϵ*, and for each a∈A a unary symbol a. Then the word $w=a_{1},\ldots ,a_{l}\in W(A)$ can be represented as value $a_{1}(\cdots (a_{l}(\epsilon ))\cdots )$ over $C_{A}$. Having this correspondence in mind, we confuse words with such values below. Furthermore, we suppose for each instruction label j=1,…,l+1 of *M* a designated constant j used to denote this label. The following lemma shows that the one-step transition relation of *M* is expressible by a predicative TRS $R_{0}^{M}$ of degree 0.

Lemma 9 *Let M be a RM with m registers. There exists a predicative tail-recursive TRS* $R_{0}^{M}$ *of degree* 0 *defining the symbols* $M_{0},M_{1},\ldots ,M_{m}$*, such that*

$$M_{0}(\;;\;j,u_{1},\ldots ,v_{m})\to_{R_{0}^{M}}j^{\prime }\;\text{and}\;M_{i}(\;;\;j,u_{1},\ldots ,u_{m})\to_{R_{0}^{M}}v_{i}\;(i=1,\ldots ,m)$$

*iff* $\langle j,u_{1},\ldots ,u_{m}\rangle \to_{M}\langle j^{\prime },v_{1},\ldots ,v_{m}\rangle$*.*

Proof Suppose $\langle j,u_{1},\ldots ,u_{m}\rangle \to_{M}\langle j^{\prime },v_{1},\ldots ,v_{m}\rangle$. For the definition of $M_{0}$, note that $j^{\prime }\neq j+1$ only if the *j*th instruction in the control of *M* is a jump instruction. In this case, $j^{\prime }$ can be determined by the left-most character of one of the values $u_{i}$ (i∈{1,…,m}). And so $j^{\prime }$ can be computed in one step using pattern-matching on the inputs $j,u_{1},\ldots ,u_{m}$ only. Similarly, for the definition of $M_{i}$ (i=1,…,m), the word $v_{i}$ is either $a(u_{i})$, *ϵ*, one of $u_{1},\ldots ,u_{m}$ or the direct subterm of $u_{i}$. Again the precise shape can be determined purely by pattern matching on the inputs $j,u_{1},\ldots ,u_{m}$.  □

Lemma 10 *Let* $f\in \mathrm{DTIME}(n^{d})$*. Then f is computed by a predicative tail-recursive TRS* $R_{f}$ *of degree d.*

Proof Suppose the function $f\;:\;W{\left(A\right)}^{k}\to W(A)$ is computed by an RM *M* in time $p(n)\in O(n^{d})$. Let c,e∈N denote constants such that $p(n)⩽c\cdot {\left|n\right|}^{d}+e$ for all n∈N. The construction of $R_{f}$ is an adaption of the TRS $R_{p}$ given in Example 9, using the TRS $R_{0}^{M}$ provided in Lemma 9 to simulate one step of the RM *M*. Let m⩾k be the numbers of registers of *M*. For function symbols $\overset{\to }{M}:=M_{0},\ldots ,M_{m}$ as provided in Lemma 9, let ${\overset{\to }{M}}^{\langle \ell \rangle }(\;;\;\overset{\to }{t})$ be the *ℓ*-*fold parallel composition* of $\overset{\to }{M}$ on terms $\overset{\to }{t}$, given by ${\overset{\to }{M}}^{\langle 0\rangle }(\;;\;\overset{\to }{t}):=\overset{\to }{t}$ and

$${\overset{\to }{M}}^{\langle \ell +1\rangle }(\;;\;\overset{\to }{t}):=M_{0}(\;;\;{\overset{\to }{M}}^{\langle \ell \rangle }(\;;\;\overset{\to }{t})),\ldots ,M_{m}(\;;\;{\overset{\to }{M}}^{\langle \ell \rangle }(\;;\;\overset{\to }{t})).$$

Observe that iterated application of Lemma 9 yields:

(1) $${\overset{\to }{M}}_{j}^{\langle \ell \rangle }(\;;\;l,u_{1},\ldots ,u_{m})\to_{R_{0}^{M}}^{⁎}v_{j}\;\;⟺\;\;(l,u_{1},\ldots ,u_{m})\to_{M}^{\ell }(l^{\prime },v_{1},\ldots ,v_{k})\;\text{for all}\;\ell ⩾1\;\text{and}\;j=1,\ldots ,m.$$

For each r=1,…,d and i=0,…,m, let $f_{r,i}$ be fresh a function symbol with 2⋅k normal and *m* safe argument positions. Let $\overset{\to }{x}:=x_{1},\ldots ,x_{k}$, $\overset{\to }{y}:=y_{1},\ldots ,y_{k}$ and $\overset{\to }{z}:=z_{0},\ldots ,z_{m}$ denote pairwise distinct variables. The TRS $R_{f}$ extends $R_{0}^{M}$ by the following rules.

$$f_{0,i}(\overset{\to }{x},\overset{\to }{y}\;;\;\overset{\to }{z})\to {\overset{\to }{M}}_{i}^{\langle c\rangle }(\;;\;\overset{\to }{z})$$

$$f_{r,i}(\overset{\to }{\epsilon },\overset{\to }{y}\;;\;\overset{\to }{z})\to z_{i}$$

$$f_{r,i}(\overset{\to }{\epsilon },a(x_{j}),\ldots ,x_{k},\overset{\to }{y}\;;\;\overset{\to }{z})\to f_{r,i}(\overset{\to }{\epsilon },x_{j},\ldots ,x_{k},\overset{\to }{y}\;;\;f_{r-1,0}(\overset{\to }{y},\overset{\to }{y}\;;\;\overset{\to }{z}),\ldots ,f_{r-1,m}(\overset{\to }{y},\overset{\to }{y}\;;\;\overset{\to }{z}))$$

$$f(\overset{\to }{x}\;;\;)\to f_{d,m}(\overset{\to }{x},\overset{\to }{x}\;;\;{\overset{\to }{M}}^{\langle e\rangle }(\;;\;1,\overset{\to }{x},\overset{\to }{\epsilon })).$$

Here the index *r* ranges over 1,…,d, the index *i* ranges over 0,…,k and a∈A. Let $\overset{\to }{u}$ and $\overset{\to }{v}$ be vectors of words of length *k*. Observe that

$$f_{r,i}(\overset{\to }{u},\overset{\to }{v}\;;\;w_{1},\ldots ,w_{k},\overset{\to }{\epsilon })\to_{R_{f}}^{⁎}{\overset{\to }{M}}_{i}^{\langle c\cdot |\overset{\to }{u}|\cdot {\left|\overset{\to }{v}\right|}^{r-1}\rangle }(\;;\;w_{1},\ldots ,w_{k},\overset{\to }{\epsilon })↓.$$

This derivation can be shown by induction on *r* and $\left|\overset{\to }{u}\right|$, in correspondence to Example 9. For words $\overset{\to }{w}=w_{1},\ldots ,w_{k}$, this thus yields

(2) $$f(\overset{\to }{w}\;;\;)\to_{R_{f}}^{⁎}{\overset{\to }{M}}_{i}^{\langle c\cdot |\overset{\to }{w}|\cdot {\left|\overset{\to }{w}\right|}^{d-1}\rangle }(\;;\;{\overset{\to }{M}}_{m}^{\langle e\rangle }(\;;\;1,\overset{\to }{w},\overset{\to }{\varepsilon }))↓={\overset{\to }{M}}_{m}^{\langle c\cdot {\left|\overset{\to }{w}\right|}^{d}+e\rangle }(\;;\;1,\overset{\to }{w},\overset{\to }{\varepsilon })↓.$$

Putting the derivations (1) and (2) together, and using that RM *M* runs in time $p(n)⩽c\cdot {\left|\overset{\to }{w}\right|}^{d}+e$ on input $w_{1},\ldots ,w_{k}$, we conclude that $〚f〛(w_{1},\ldots ,w_{k})=f(w_{1},\ldots ,w_{k})$ holds.

Observe that the precedence ⩾ of $R_{f}$ on defined symbols is given by

$$f>f_{d,0},\ldots ,f_{d,m}>\cdots >f_{0,0},\ldots ,f_{0,m}>M_{0},\ldots ,M_{m},$$

where only the symbols $f_{r,i}$ for r>0 are recursive in $R_{f}$. In particular, the recursion depth of $R_{f}$ is thus *d*. It is also not difficult to see that $R_{f}$ is predicative recursive. As $R_{f}$ is tail-recursive, the lemma follows.  □

### 6.2 Predicative tail-recursive TRSs of degree *d* are sound for $\mathrm{DTIME}(n^{d})$

We now show the converse of Lemma 10. Fix a predicative tail-recursive TRS R of degree *d*. Call a function symbol *monadic* if its arity is at most one. Suppose all constructors of R are monadic and consider a defined symbol *f* in R. We show that the function 〚f〛 computed by R can be implemented on an RM $M_{f}$, operating in time $O(n^{d})$. The restriction to monadic constructors allows us to identify values of R with words over the alphabet $A_{C}$, which contains for every constructor $a_{i}\in C$ a distinct letter $a_{i}$. We use the word $c_{1},\ldots ,c_{l}$ to denote the value $c_{1}(c_{2}(\ldots c_{l-1}(c_{l})\ldots ))$. Having this correspondence in mind, we again confuse words with values.

To ease presentation, we first consider the sub-case where R is *simple*. Here a rule $f(\overset{\to }{u}\;;\;\overset{\to }{v})\to r$ is called *simple* if *r* is a constructor term or $r=g(\overset{\to }{w}\;;\;h_{1}(\overset{\to }{u}\;;\;\overset{\to }{v}),\ldots ,h_{k}(\overset{\to }{u}\;;\;\overset{\to }{v}))$ where *g* is either a defined or a constructor symbol and $h_{1},\ldots ,h_{k}\in D$. Furthermore R is called *simple* if all its rules are simple.

Lemma 11 *If* R *is simple, then* $〚f〛\in \mathrm{DTIME}(n^{\mathrm{rd}(f)})$ *for every defined symbol f from* R*.*

Proof For each defined symbol *f* in R with *k* normal and *l* safe arguments, we define a corresponding RM $M_{f}$ with input registers ${\overset{\to }{x}}_{f}=x_{1}^{f},\ldots ,x_{k+l}^{f}$ and output variable $z^{f}$. On input $\overset{\to }{u}=u_{1},\ldots ,u_{k}$ and $\overset{\to }{v}=v_{1},\ldots ,v_{l}$ the RMs $M_{f}$ run in time $O({\left|\overset{\to }{u}\right|}^{\mathrm{rd}(f)})$. To simplify the presentation, we first suppose that the precedence of R is strict on defined symbols, i.e. f∼g for f,g∈D implies f=g. The construction is by induction on the rank *p* of *f*, the bound is proven by induction on *p* and side induction on |u|. Suppose the input registers ${\overset{\to }{x}}_{f}$ hold the values $\overset{\to }{u},\overset{\to }{v}$.

First observe that $M_{f}$ is able to determine in constant time (depending only on R) the (unique) rewrite rule applicable to $f(\overset{\to }{u}\;;\;\overset{\to }{v})$. Since there are only a constant number of rules in R, it suffice to realise that the time required for pattern matching depends only on R. To this end, suppose we want to match $f(\overset{\to }{u}\;;\;\overset{\to }{v})$ against the left hand-side $f(\overset{\to }{l_{n}}\;;\;\overset{\to }{l_{s}})\to r\in R$. Due to linearity, $M_{f}$ can match the arguments $\overset{\to }{u},\overset{\to }{v}$ against $\overset{\to }{l_{n}},\overset{\to }{l_{s}}$ individually. For this, the RM $M_{f}$ just has to copy sequentially each argument $w\in \overset{\to }{u},\overset{\to }{v}$ to a temporary register, $w_{i}$ can then be matched against the corresponding argument $l_{i}\in \overset{\to }{l_{n}},\overset{\to }{l_{s}}$ using a constant number of jump and delete instructions.

Once the applicable rewrite rule has been identified, the RM $M_{f}$ can proceed according to its right-hand side as follows. If $f(\overset{\to }{u}\;;\;\overset{\to }{v})$ rewrites in one step to a value, say *w*, then w=C[xσ] for some constructor context *C* and substitution σ:V→T(C). Then some input register $x_{i}\in {\overset{\to }{x}}_{f}$ holds the word $C^{\prime }[x\sigma ]$. Notice that the contexts *C* and $C^{\prime }$ depend only on the applied rewrite rule. Hence $M_{f}$ can provide the result *w* in register $z_{f}$ in constant time. Thus suppose $f(\overset{\to }{u}\;;\;\overset{\to }{v})$ does not rewrite to a value in one step. Since R is simple

$$f(\overset{\to }{u}\;;\;\overset{\to }{v})\to_{R}g(w_{1},\ldots ,w_{m}\;;\;h_{1}(\overset{\to }{u}\;;\;\overset{\to }{v}),\ldots ,h_{n}(\overset{\to }{u}\;;\;\overset{\to }{v}))\;\text{where}\;h_{1},\ldots ,h_{n}\in D.$$

As R is predicative recursive, $f>h_{j}$ holds for all j=1,…,n. Furthermore, either f>g or f=g holds by our assumption that ⩾ is strict on defined symbols. In both cases, order constraints on normal arguments give $f(\overset{\to }{u}\;;\;\overset{\to }{v}){▷}^{\text{n}}{/}_{∼}w_{i}$ (i=1,…,m), i.e.  some input register holds a superterm of $w_{i}$. The RM $M_{f}$ can prepare the arguments $w_{i}$ in dedicated registers $x_{i}^{g}$ for all i=1,…,m in constant time. By induction hypothesis, there exist RMs $M_{h_{j}}$ (j=1,…,n) that on input registers ${\overset{\to }{x}}_{h_{j}}$ initialised by $\overset{\to }{u},\overset{\to }{v}$, compute the value $〚h_{j}〛(\overset{\to }{u}\;;\;\overset{\to }{v})$ in time $O({\left|\overset{\to }{u}\right|}^{\mathrm{rd}(h_{j})})$. The RM $M_{f}$ can use these machines as sub-procedures (cf. [34]) in order to compute $〚h_{j}〛(\overset{\to }{u}\;;\;\overset{\to }{v})$ (j=1,…,n). Overall this requires at most $O({\left|\overset{\to }{u}\right|}^{d})$ steps, where $d:=\max\{\mathrm{rd}(h_{1}),\ldots ,\mathrm{rd}(h_{n})\}⩽\mathrm{rd}(f)$ is the maximal recursion depth of the defined symbols $h_{i}$. The interesting case is now when g∈D. We analyse the cases f>g and f=g independently.

If f>g then as before we can use a machine $M_{g}$ given by induction hypothesis that computes $〚g〛(\overset{\to }{w}\;;\;〚h_{1}〛(\overset{\to }{u}\;;\;\overset{\to }{v}),\ldots ,〚h_{n}〛(\overset{\to }{u}\;;\;\overset{\to }{v}))=〚f〛(\overset{\to }{u}\;;\;\overset{\to }{v})$ where $\overset{\to }{w}:=w_{1},\ldots ,w_{m}$ in time $O({\left|\overset{\to }{w}\right|}^{\mathrm{rd}(g)})$, from the already initialised input registers ${\overset{\to }{x}}^{g}$. As a consequence of the order constraints on R we see $\left|w_{i}|⩽\max\{|u_{j}||u_{j}\in \overset{\to }{u}\right\}$ for all i=1,…,m. Thus $\left|w|⩽m\cdot |\overset{\to }{u}\right|$, and hence overall the procedure takes time $O({\left|\overset{\to }{u}\right|}^{d})+O({\left|\overset{\to }{w}\right|}^{\mathrm{rd}(g)})\subseteq O({\left|\overset{\to }{u}\right|}^{\mathrm{rd}(f)})$. For the inclusion we employ d⩽rd(f) and rd(g)⩽rd(f) as given by the assumptions.

Otherwise f=g, hence *f* is recursive. Recall that ${>}_{{\mathrm{spop}}_{\mathrm{ps}}^{⁎}}$ collapses to the subterm relation (modulo equivalence) on values. From the order constraint on normal arguments $\langle \overset{\to }{u}\rangle {>}_{{\mathrm{spop}}_{\mathrm{ps}}^{⁎}}\langle \overset{\to }{w}\rangle$ it is thus not difficult to derive $\left|\overset{\to }{w}|<|\overset{\to }{u}\right|$. Recall $d=\max\{\mathrm{rd}(h_{1}),\ldots ,\mathrm{rd}(h_{n})\}<\mathrm{rd}(f)$ since *f* is recursive. Thus it follows that ${\left|\overset{\to }{u}\right|}^{d}+{\left|\overset{\to }{w}\right|}^{\mathrm{rd}(f)}⩽{\left|\overset{\to }{u}\right|}^{\mathrm{rd}(f)}+1$. Using the side induction hypothesis we conclude that $M_{f}$ operates in time $O({\left|\overset{\to }{u}\right|}^{d})+O({\left|\overset{\to }{w}\right|}^{\mathrm{rd}(f)})=O({\left|\overset{\to }{u}\right|}^{\mathrm{rd}(f)})$ overall. We conclude this final case.

To lift the assumption on the precedence, suppose $\left\{f_{1},\ldots ,f_{\ell }\right\}$ is the set of all function symbols equivalent to f∈D, i.e., $f_{1},\ldots ,f_{\ell }$ are defined by mutual recursion. Since this class is finite, one can store *i* (for i=1,…,ℓ) in a dedicated register of $M_{f}$, say *r*. Although more tedious, it is not difficult to see that the above construction can then be altered, so that $M_{f}$ computes $f_{\langle r\rangle }(\overset{\to }{u}\;;\;\overset{\to }{v})$ on input $\overset{\to }{u},\overset{\to }{v}$.  □

We now remove the restriction that R is simple. For that we define the relation ⇝ on TRSs as follows. Let $h_{1},\ldots ,h_{k}$ be fresh symbols not appearing in R. Then

$$R⊎\{f({\overset{\to }{u}}_{f}\;;\;{\overset{\to }{v}}_{f})\to g({\overset{\to }{u}}_{g}\;;\;t_{1},\ldots ,t_{k})\}⇝R\cup \{f({\overset{\to }{u}}_{f}\;;\;{\overset{\to }{v}}_{f})\to g({\overset{\to }{u}}_{g}\;;\;h_{1}({\overset{\to }{u}}_{f}\;;\;{\overset{\to }{v}}_{f}),\ldots ,h_{k}({\overset{\to }{u}}_{f}\;;\;{\overset{\to }{v}}_{f}))\}\cup \{h_{i}({\overset{\to }{u}}_{f}\;;\;{\overset{\to }{v}}_{f})\to t_{i}|i=1,\ldots ,k\},$$

provided the *transformed rule* $f({\overset{\to }{u}}_{f}\;;\;{\overset{\to }{v}}_{f})\to g({\overset{\to }{u}}_{g}\;;\;t_{1},\ldots ,t_{k})$ is not already simple. The relation ⇝ enjoys following properties.

Lemma 12

1. *The relation* ⇝ *is well-founded.*
2. *If* R⇝S *then* $\to_{R}\subseteq \to_{S}^{+}$*.*
3. *Let* R *be a predicative tail-recursive TRS of degree d that uses only monadic constructors. If* R⇝S *then* S *enjoys the same properties.*

Proof Let $\left\|R\|:=\sum_{r\in R}|r\right|$, where R={r|l→r∈Ris not a simple rule}. Then an infinite chain $R_{1}⇝R_{2}⇝\cdots$ translates into an infinite descend $\|R_{1}\|>\|R_{2}\|>\cdots$. Hence property 1 follows.

Suppose now R⇝S. For property 2, consider a rewrite step $C[f({\overset{\to }{u}}_{f}\sigma \;;\;{\overset{\to }{v}}_{f}\sigma )]\to_{R}C[g({\overset{\to }{u}}_{g}\sigma \;;\;t_{1}\sigma ,\ldots ,t_{k}\sigma )]$ using a transformed rule $f({\overset{\to }{u}}_{f}\;;\;{\overset{\to }{v}}_{f})\to g({\overset{\to }{u}}_{g}\;;\;t_{1},\ldots ,t_{k})\in R$. Then

$$C[f({\overset{\to }{u}}_{f}\sigma \;;\;{\overset{\to }{v}}_{f}\sigma )]\to_{S}C[g({\overset{\to }{u}}_{g}\sigma \;;\;h_{1}({\overset{\to }{u}}_{f}\sigma \;;\;{\overset{\to }{v}}_{f}\sigma ),\ldots ,h_{k}({\overset{\to }{u}}_{f}\sigma \;;\;{\overset{\to }{v}}_{f}\sigma ))]\to_{S}^{k}C[g({\overset{\to }{u}}_{g}\sigma \;;\;t_{1}\sigma ,\ldots ,t_{k}\sigma )],$$

simulates the considered step. So clearly $\to_{R}\subseteq \to_{S}^{+}$ follows.

Finally consider property 3, and consider a TRS S such that R⇝S. Let $f({\overset{\to }{u}}_{f}\;;\;{\overset{\to }{v}}_{f})\to g({\overset{\to }{u}}_{g}\;;\;t_{1},\ldots ,t_{k})$, denote the rule which is replaced by

$$f({\overset{\to }{u}}_{f}\;;\;{\overset{\to }{v}}_{f})\to g({\overset{\to }{u}}_{g}\;;\;h_{1}({\overset{\to }{u}}_{f}\;;\;{\overset{\to }{v}}_{f}),\ldots ,h_{k}({\overset{\to }{u}}_{f}\;;\;{\overset{\to }{v}}_{f}))\;\text{and}\;h_{i}({\overset{\to }{u}}_{f}\;;\;{\overset{\to }{v}}_{f})\to t_{i}\;(i=1,\ldots ,k).$$

Let ⩾ denote the precedence underlying R, and ⊒ the precedence underlying the simplified TRS S. Notice that ⊒ is an extension of ⩾, which collapses to ⩾ on the signature F of R. As the freshly introduced symbols $h_{i}$ are not recursive, the recursion depth of every symbol h∈F is preserved by the transformation.

It is obvious that when R is tail-recursive, so is S. It thus remains to verify that S is oriented by the order ${⊐}_{{\mathrm{spop}}_{\mathrm{ps}}^{⁎}}$. Since ⩾⊆⊒, and as a consequence ${>}_{{\mathrm{spop}}_{\mathrm{ps}}^{⁎}}\subseteq {⊒}_{{\mathrm{spop}}_{\mathrm{ps}}^{⁎}}$, it suffices to show that the orientation $f({\overset{\to }{u}}_{f}\;;\;{\overset{\to }{v}}_{f}){>}_{{\mathrm{spop}}_{\mathrm{ps}}^{⁎}}g({\overset{\to }{u}}_{g}\;;\;t_{1},\ldots ,t_{k})$ of the replaced rule implies

(3) $$f({\overset{\to }{u}}_{f}\;;\;{\overset{\to }{v}}_{f}){⊐}_{{\mathrm{spop}}_{\mathrm{ps}}^{⁎}}g({\overset{\to }{u}}_{g}\;;\;h_{1}({\overset{\to }{u}}_{f}\;;\;{\overset{\to }{v}}_{f}),\ldots ,h_{k}({\overset{\to }{u}}_{f}\;;\;{\overset{\to }{v}}_{f}))$$

(4) $$h_{i}({\overset{\to }{u}}_{f}\;;\;{\overset{\to }{v}}_{f}){⊐}_{{\mathrm{spop}}_{\mathrm{ps}}^{⁎}}t_{i}\;(i=1,\ldots ,k).$$

We perform case analysis on the assumption.

Suppose first $f({\overset{\to }{u}}_{f}\;;\;{\overset{\to }{v}}_{f}){>}_{{\mathrm{spop}}_{\mathrm{ps}}^{⁎}}^{\langle 1\rangle }g({\overset{\to }{u}}_{g}\;;\;t_{1},\ldots ,t_{k})$ holds. Note that by the shape of left-hand sides in R and definition of the precedence, *g* is a constructor in the considered case. In particular *g* admits only safe argument positions. Thus $f({\overset{\to }{u}}_{f}\;;\;{\overset{\to }{v}}_{f}){⊐}_{{\mathrm{spop}}_{\mathrm{ps}}^{⁎}}^{\langle 2\rangle }g({\overset{\to }{u}}_{g}\;;\;h_{1}({\overset{\to }{u}}_{f}\;;\;{\overset{\to }{v}}_{f}),\ldots ,h_{k}({\overset{\to }{u}}_{f}\;;\;{\overset{\to }{v}}_{f}))$ holds using $f({\overset{\to }{u}}_{f}\;;\;{\overset{\to }{v}}_{f}){⊐}_{{\mathrm{spop}}_{\mathrm{ps}}^{⁎}}^{\langle 2\rangle }h_{i}({\overset{\to }{u}}_{f}\;;\;{\overset{\to }{v}}_{f})$ (i=1,…,k). This concludes (3). The assumption give also $f({\overset{\to }{u}}_{f}\;;\;{\overset{\to }{v}}_{f})▷{/}_{∼}t_{i}$ for all i=1,…,k, thus $h_{i}({\overset{\to }{u}}_{f}\;;\;{\overset{\to }{v}}_{f}){⊐}_{{\mathrm{spop}}_{\mathrm{ps}}^{⁎}}^{\langle 1\rangle }t_{i}$ holds and we conclude (4).

Finally suppose that $f({\overset{\to }{u}}_{f}\;;\;{\overset{\to }{v}}_{f}){>}_{{\mathrm{spop}}_{\mathrm{ps}}^{⁎}}g({\overset{\to }{u}}_{g}\;;\;t_{1},\ldots ,t_{k})$ follows by ${>}_{{\mathrm{spop}}_{\mathrm{ps}}^{⁎}}^{\langle 2\rangle }$ either or ${>}_{{\mathrm{spop}}_{\mathrm{ps}}^{⁎}}^{\langle 3\rangle }$. Using the order constraint $f({\overset{\to }{u}}_{f}\;;\;{\overset{\to }{v}}_{f}){>}_{{\mathrm{spop}}_{\mathrm{ps}}^{⁎}}h_{i}({\overset{\to }{u}}_{f}\;;\;{\overset{\to }{v}}_{f})$ for all i=1,…,k, we see that (3) follows by ${⊐}_{{\mathrm{spop}}_{\mathrm{ps}}^{⁎}}^{\langle 2\rangle }$ or ${⊐}_{{\mathrm{spop}}_{\mathrm{ps}}^{⁎}}^{\langle 3\rangle }$ respectively. For Eq. (4), observe that since R is tail-recursive, the assumption gives $f({\overset{\to }{u}}_{f}\;;\;{\overset{\to }{v}}_{f}){>}_{{\mathrm{spop}}_{\mathrm{ps}}^{⁎}}t_{i}$ (i=1,…,k) using only applications of ${>}_{{\mathrm{spop}}_{\mathrm{ps}}^{⁎}}^{\langle 1\rangle }$ and ${>}_{{\mathrm{spop}}_{\mathrm{ps}}^{⁎}}^{\langle 2\rangle }$. Repeating these proofs, but employing $h_{i}⊐g$ instead of f>g yields a proof of (4).  □

Lemma 13 *Let* R *be a predicative tail-recursive TRS of degree d, and suppose all constructors are monadic. Then* $〚f〛\in \mathrm{DTIME}(n^{\mathrm{rd}(f)})$ *for every defined symbol f from* R*.*

Proof Let S be a ⇝-normal form of our analysed TRS R. Then S is simple as otherwise ⇝-reducible. Using the assumptions on R, Lemma 12 yields that S satisfies the preconditions of Lemma 11. Moreover, it shows that S computes all functions computed by R. We conclude by Lemma 11.  □

### 6.3 Predicative tail-recursive TRSs of degree *d* characterise $\mathrm{DTIME}(n^{d})$

By Lemma 10 and Lemma 13, we obtain following correspondence.

Theorem 6 *For each* d∈N*, the following classes of functions are equivalent:*

1. *The class of functions computed by predicative tail-recursive TRSs of degree d, using only monadic constructors.*
2. *The class* $\mathrm{DTIME}(n^{d})$ *of functions computed by register machines operating in time* $O(n^{d})$*.*

This theorem is closely connected to the recursion-theoretic characterisation of the polytime computable functions provided by Leivant [12], and the one of Marion [13]. Leivant uses *ramified recurrence schemes* to impose data tiering on functions defined by recursive means. Restricted to word algebras and two tiers, a function *f* in Leivant's class belongs to $\mathrm{DTIME}(n^{d})$, where *d* corresponds to the number of nested recursive definitions in *f*. Vice verse, any function in $\mathrm{DTIME}(n^{d})$ is expressible in Leivant's class using two tiers, and maximal *d* nested recursive definitions. Hence there is a precise correspondence between the functions *f* defined in Leivant's class based on *d* nested recursive definitions, and the functions definable by predicative recursive TRS of degree *d*. Syntactically, the restriction to two tiers in Leivant's class results in a composition scheme conceptually similar to weak safe composition. Substitution is only allowed on arguments not used for recursion.

Our result as well as Leivant's characterisation, relies on recursion schemes that go beyond primitive recursion with data tiering. Leivant allows recursive definitions by *simultaneous recursion*. We note that in our context, simultaneous recursion cannot be permitted. In general, such an extension would invalidate Theorem 1 and Theorem 2 respectively. Instead, we resort to parameter substitution, which is essential for our completeness result. Still simultaneous recursion can be reduced to primitive recursion, preserving the data tiering principle underlying [12]. However, this program transformation relies on a form of tupling, and does not preserve the number of nestings of recursive definitions. In our context, parameter substitution can be eliminated in recursive definitions in a similar spirit, at the expense of the depth of recursion.

Restoring to *strict ramified recurrence schemes*, Marion [13] provides a fine-grained characterisation of $\mathrm{DTIME}(n^{d})$ in the spirit of Leivant's characterisation and our result above. The underlying strict ramification principle requires that each recursive definition increases the tier of an input. As a consequence, the exponent *d* is reflected in the maximal level of an input tier. Crucial here again is the restriction to a composition scheme akin to our weak form of composition.

In Marion's characterisation, functions can return multiple values. As a consequence, the simulation of register machine computations requires neither simultaneous recursion nor similar concepts. It is not difficult to show that a modification of our computational model, which accounts for multi-valued functions, allows the completeness result given in Lemma 10 even if we disallow parameter substitution. We feel however that such a modification, tailored specifically to register machines, introduces a rather ad-hoc flavor to our formulation of computation by TRSs.

## 7 Examples and experimental evaluation

We briefly contrast the orders ${\text{sPOP}}^{⁎}$ and ${\text{sPOP}}_{\mathrm{PS}}^{⁎}$ to its predecessor ${\text{POP}}^{⁎}$ [9], Marion's LMPO [6], as well as interpretation methods found in state-of-the-art complexity provers. Furthermore, we present experimental results.

##### Lightweight multiset path order and polynomial path orders

The order ${\text{sPOP}}^{⁎}$ forms a restriction of ${\text{POP}}^{⁎}$ and LMPO, whereas the latter two orders are incomparable in general. In contrast to the family of polynomial path orders, LMPO allows multiple recursive calls in right-hand sides. As clarified in the next example, extending our methods would invalidate the corresponding main results (Theorem 1 and Theorem 2 respectively).

Example 10 The TRS $R_{\mathrm{bin}}$ is given by the following rules:

bin(x,0)→s(0)bin(0,s(y))→0bin(s(x),s(y))→+(bin(x,s(y)),bin(s(x),y)).

For a precedence ⩾ that fulfills bin>s and bin>+ we obtain that $R_{\mathrm{bin}}$ is compatible with LMPO. This TRS can however neither be handled by ${\text{sPOP}}^{⁎}$ nor ${\text{sPOP}}_{\mathrm{PS}}^{⁎}$. It is straightforward to verify that the family of terms $\mathrm{bin}(s^{n}(0),s^{m}(0))$ admits derivations whose length grows exponentially in *n*. Still the underlying function can be proven polynomial, essentially relying on memoisation techniques [6].

On the other hand, ${\text{POP}}^{⁎}$ integrates a multiset status. In contrast, both LMPO and ${\text{sPOP}}^{⁎}$  are restricted to product status.

Example 11 Consider the following one-ruled TRS $R_{\mathrm{levy}}$ originally stemming from Jean-Jaques Lévy^4^:

f(g(;x),y;y)→g(;f(x,x;y)).

Polynomial runtime complexity of this system can be shown by ${\text{POP}}^{⁎}$. The system is neither compatible with an instance of LMPO nor ${\text{sPOP}}^{⁎}$, because the product of arguments to f cannot be ordered.

However, the system becomes orientable with an instance of ${\text{sPOP}}_{\mathrm{PS}}^{⁎}$, if we make also the second argument of f safe. Observe that $f(g(\;;\;x)\;;\;y,y){>}_{{\mathrm{spop}}_{\mathrm{ps}}^{⁎}}^{\langle 3\rangle }f(x\;;\;x,y)$ holds, using $\langle g(\;;\;x)\rangle {>}_{{\mathrm{spop}}_{\mathrm{ps}}^{⁎}}\langle x\rangle$, $f(g(\;;\;x)\;;\;y,y){>}_{{\mathrm{spop}}_{\mathrm{ps}}^{⁎}}^{\langle 1\rangle }x$ as well as $f(g(\;;\;x)\;;\;y,y){>}_{{\mathrm{spop}}_{\mathrm{ps}}^{⁎}}^{\langle 1\rangle }y$. From this, one application of ${>}_{{\mathrm{spop}}_{\mathrm{ps}}^{⁎}}^{\langle 2\rangle }$ orients the rule. Since f is the only recursive symbol, Theorem 2 shows that the runtime complexity of $R_{\mathrm{levy}}$ is at most linear.

Even though ${\text{sPOP}}^{⁎}$ forms a restriction of ${\text{POP}}^{⁎}$ and LMPO, its extension by parameter substitution is incomparable to LMPO and ${\text{POP}}^{⁎}$. Consider the following TRS.

Example 12 The TRS $R_{\mathrm{add}}$ consists of the following rules.^5^

+(0;y)→y+(s(;x);y)→s(;+(x;y))+(s(;x);y)→+(x;s(;y))

Due to the last rule, the TRS $R_{\mathrm{add}}$ is neither compatible with ${\text{sPOP}}^{⁎}$, ${\text{POP}}^{⁎}$ nor LMPO. The system is however compatible with the instance ${>}_{{\mathrm{spop}}_{\mathrm{ps}}^{⁎}}$ of ${\text{sPOP}}_{\mathrm{PS}}^{⁎}$  as induced by the precedence underlying $R_{\mathrm{add}}$ and separation of argument positions indicated in the rules. The degree of recursion of $R_{\mathrm{add}}$ is one. The runtime complexity of $R_{\mathrm{add}}$ is inferred to be linear by Theorem 2.

We remark that ${\text{POP}}^{⁎}$ can be extended by parameter substitution [9]. Unless PSPACE=P, this extension does however not carry over to LMPO, without sacrificing polytime computability. For instance, the natural extension of LMPO by parameter substitution can handle Example 36 from [11]. This example encodes the PSPACE complete problem of quantifier elimination on quantified Boolean formulas.

##### Polynomial and matrix interpretations

Small polynomial path orders are in general incomparable to interpretation methods, notably *matrix* [37] and *polynomial interpretations* [38]. These are the most frequently used base techniques in complexity tools nowadays. A *polynomial interpretation* is an F-algebra [29] A with carrier N, where interpretations $f_{A}\;:\;N^{k}\to N$ (for every *k*-ary function symbol *f*) are monotone polynomials. A TRS R is *compatible* with a polynomial interpretation, aka *polynomially terminating*, if for every rule l→r∈R, under any assignment the left-hand side *l* is interpreted in A larger than the interpretation of the right-hand side *r*. We say that a polynomial interpretation A *induces* polynomial runtime complexity if the interpretation of every basic term is bounded by a polynomial in the size of *s*. For such an interpretation A compatible with R, the runtime complexity of R is bounded by a polynomial. *Additive polynomial interpretations* [10], where all *constructors c* are interpreted by *additive polynomials* $c_{A}(x_{1},\ldots ,x_{k})=\delta +\sum_{i=1}^{k}x_{i}$ (δ∈N) induce polynomial runtime complexity.

Not every polynomially terminating TRS is predicative recursive, even if only additive interpretations are employed. Vice verse, not every predicative recursive TRS is polynomially terminating so that the underlying interpretation induces polynomial runtime complexity. This is clarified in the next two examples.

Example 13 The one-ruled TRS {f(c(x))→f(d(x))} is polynomially terminating, using interpretations $c_{A}(x)=x+1$ and $f_{A}(x)=d_{A}(x)=x$, but it is not compatible with any of the above mentioned restrictions of recursive path orders.

Example 14 Consider the predicative tail-recursive TRS $R_{\mathrm{btree}}$, which consists of the following two rewrite rules:

f(0;y)→yf(s(;x);y)→f(x;c(;y,y)).

Suppose this rewrite system is compatible with a polynomial interpretation A. Consider the reduction of a basic term $s_{n}:=f(s^{n}(\;;\;0)\;;\;s(\;;\;0))$ for n∈N. This yields a binary tree $v_{n}$ of height *n*, with leafs s(;0). Observe that by monotonicity, $c_{A}(x,y)⩾x+y$ holds. Note that orientation requires that $s_{A}(x)>x$. As a consequence, the interpretation of terms $v_{n}$ grows exponentially in *n*. As by compatibility the interpretations of terms necessarily decrease during reduction, it follows that A does not induce polynomial runtime complexity.

A *matrix interpretation* A is similar to a polynomial interpretation, but the underlying carrier of the F-algebra is $N^{d}$ (d⩾1), and interpretation functions are of shape $f_{A}({\overset{\to }{x}}_{1},\ldots ,{\overset{\to }{x}}_{k})=F_{1}\cdot {\overset{\to }{x}}_{1}+\cdots +F_{k}\cdot {\overset{\to }{x}}_{k}+f$. Here $F_{i}$ (i=1,…,k) denote matrices of size d×d, and *f* is vector over N. The notions of compatibility and induced polynomial complexity carry over naturally from polynomial interpretations. As for (additive) polynomial interpretations it can be shown that matrix interpretations are incompatible to small path orders. This is clarified in the next example.

Example 15 **Continued from Examples 2 and 13**

Reconsider the predicative recursive TRS $R_{\mathrm{arith}}$ from Example 1. This system cannot be shown compatible with matrix interpretations. Intuitively this holds due to the linear form of matrix interpretations. The interpretation of a basic term ×(x,y;) has to be a non-linear expression in both *x* and *y*.

Vice verse, the (additive) polynomial interpretation given in Example 13 turns naturally into a matrix interpretation compatible with the one-ruled TRS depicted in Example 13. On the other hand, this TRS is not predicative recursive.

Our final example shows that even in cases where semantic methods apply, order-based techniques might deduce a tighter bound.

Example 16 **Continued from Example 11**

While the TRS $R_{\mathrm{levy}}$ given in Example 11 can be handled with semantic methods, the polynomial interpretations can only verify a quadratic upper bound. To the contrary, ${\text{sPOP}}_{\mathrm{PS}}^{⁎}$  can verify the (non-optimal) linear bound.

##### Experimental assessment

The small polynomial path order ${\text{sPOP}}^{⁎}$ gives rise to a new, fully automatic, syntactic method for polynomial runtime complexity analysis. We have implemented this technique in our complexity tool  [2]. In particular the complexity proofs above have been obtained automatically with .

In order to further test the viability of small polynomial path orders, we performed experiments on the relative power of ${\text{sPOP}}^{⁎}$ (respectively ${\text{sPOP}}_{\mathrm{PS}}^{⁎}$) with respect to LMPO [6], ${\text{POP}}^{⁎}$ [8] and interpretations [10,37] suited to polynomial complexity analysis. Experiments were conducted with  version 2.0,^6^ on a machine with 8 Dual-Core Opteron^™^ 885 processors (2.6 GHz). We abort  if a complexity certificate could not be found within 10 seconds. We selected two data-sets: data-set **TC** constitutes of 597 terminating constructor TRSs and data-set **TCO**, containing 290 examples, resulting from restricting test-suite **TC** to orthogonal systems.^7^

Table 1 summarises the results obtained on data-sets **TC**  and **TCO**.^8^ On the larger benchmark **TC**, the total of 39 examples drawn in column ${\text{sPOP}}^{⁎}$ are necessarily a subset of the 54 examples compatible with LMPO, and also the 43 examples compatible with ${\text{POP}}^{⁎}$. Note that LMPO induces only exponential bounded runtime complexity. On three examples, including the TRS $R_{\mathrm{bin}}$ depicted in Example 10, this bound is indeed tight. Whereas ${\text{POP}}^{⁎}$ can only give an unspecified polynomial bound, ${\text{sPOP}}^{⁎}$ assesses the complexity of compatible systems between constant and cubic. Thus ${\text{sPOP}}^{⁎}$ brings about a significant increase in precision, accompanied with only minor decrease in power. This assessment remains true, if we consider the smaller benchmark set **TCO**. Parameter substitution increases the analytic power of ${\text{POP}}^{⁎}$ on test-suite **TC** from 39 to 54 examples. From the 15 new examples, 13 examples are neither compatible with LMPO  nor ${\text{POP}}^{⁎}$.

The last two columns in Table 1 indicate the strength of semantic techniques and their combination with ${\text{sPOP}}^{⁎}$ (column SEM+${\text{sPOP}}_{\mathrm{PS}}^{⁎}$). In column SEM we employed matrix interpretations [37] (dimension 1 to 3) as well as additive polynomial interpretations [10] (degrees 2 and 3). Here we make use of the modular combination technique proposed by Zankl and Korp [39] to combine the interpretation techniques. Coefficients, respectively entries in coefficients, range up to 7. To ensure that matrix interpretations induce polynomial runtime complexity, we resort to the non-trivial criteria found in [17]. Column SEM+${\text{sPOP}}_{\mathrm{PS}}^{⁎}$ corresponds to column SEM, where ${\text{sPOP}}_{\mathrm{PS}}^{⁎}$ is additionally integrated.

It is immediate that syntactic techniques alone cannot compete with the expressive power of interpretations. If we consider the total number of compatible systems only, semantic techniques are roughly twice as powerful as the strongest syntactic technique (${\text{sPOP}}_{\mathrm{PS}}^{⁎}$). Still, the syntactic techniques proposed in this work provide a fruitful addition to the interpretation method. Contrasting columns SEM and SEM+${\text{sPOP}}_{\mathrm{PS}}^{⁎}$, not only the total number of certified systems, but also the precision of the obtained certificates, is increased by the addition of ${\text{sPOP}}_{\mathrm{PS}}^{⁎}$. Note also the slight decrease in execution time.

## 8 Conclusion

We propose a new order, the small polynomial path order ${\text{sPOP}}^{⁎}$, together with its extension ${\text{sPOP}}_{\mathrm{PS}}^{⁎}$ to *parameter substitution*. Based on ${\text{sPOP}}^{⁎}$, we delineate a class of rewrite systems, dubbed systems of predicative recursion of degree *d*, such that for rewrite systems in this class we obtain that the runtime complexity lies in $O(n^{d})$. Exploiting the control given by the degree of recursion, we establish a novel characterisation of the functions computable in time $O(n^{d})$, on *register machines* via the small polynomial path order ${\text{sPOP}}_{\mathrm{PS}}^{⁎}$.

Thus small polynomial path orders induce a new order-theoretic characterisation of the class of polytime computable functions. This order-theoretic characterisation enables a fine-grained control of the complexity of functions in relation to the number of nested applications of recursion. One the other hand, small polynomial path orders provide a novel *syntactic*, and very fast, criteria to automatically establish polynomial runtime complexity of a given TRS. The latter criteria extend the state of the art in runtime complexity analysis as it can be more precise or more efficient than previously known techniques.

## Figures and Tables

**Table 1:** Number of oriented problems and average execution times (seconds) on data-sets **TC** and **TCO**.

		LMPO	${\text{POP}}^{⁎}$	${\text{sPOP}}^{⁎}$	${\text{sPOP}}_{\mathrm{PS}}^{⁎}$	SEM	SEM+${\text{sPOP}}_{\mathrm{PS}}^{⁎}$
**TC**	O(1)	—	—	9/0.13	9/0.13	—	3/0.12
O(n)	—	—	23/0.16	37/0.21	83/0.73	89/0.70
$O(n^{2})$	—	—	6/0.22	7/0.23	20/2.17	17/1.84
$O(n^{3})$	—	—	1/0.58	1/0.62	—	1/6.66
$\underset{k\in N}{⋃}O(n^{k})$	—	43/0.12	—	—	—	—
Compatible	54/0.14	43/0.12	39/0.17	54/0.21	103/1.01	110/0.91
Incompatible	543/0.25	554/0.25	558/0.24	543/0.25	25/4.48	25/4.54
Timeout	—	—	—	—	469/10.0	462/10.0

**TCO**	O(1)	—	—	5/0.12	5/0.12	—	3/0.12
O(n)	—	—	14/0.15	19/0.18	44/0.84	45/0.78
$O(n^{2})$	—	—	4/0.20	4/0.21	13/2.04	11/1.94
$\underset{k\in N}{⋃}O(n^{k})$	—	24/0.11	—	—	—	—
Compatible	29/0.13	24/0.11	23/0.15	54/0.17	57/1.11	59/0.96
Incompatible	261/0.13	266/0.17	267/0.17	702/0.17	8/4.36	8/4.29
Timeout	—	—	—	—	225/10.0	223/10.0

## Footnotes

1 http://termcomp.uibk.ac.at/.

2 The restriction is not necessary, but simplifies our presentation, compare [9].

3 As in [9] it is possible to adopt nondeterministic semantics, dropping orthogonality.

4 This is Example 2.59 in Steinbach and Kühler's collection of TRSs [36].

5 This is Example 2.09 in Steinbach and Kühler's collection of TRSs [36].

6 Available from http://cl-informatik.uibk.ac.at/software/tct/.

7 The test-suites are taken from the Termination Problem Database (TPDB), version 8.0; http://termcomp.uibk.ac.at.

8 Full experimental evidence is provided under http://cl-informatik.uibk.ac.at/software/tct/experiments/spopstar-ICC.
